# Supplementary material for: Clinical Efficacy and Safety of Chinese Herbal Medicine in the Treatment of Uremic Pruritus: A Meta-Analysis of Randomized Controlled Trials
Source: Pharmaceuticals (Basel). 2022 Oct 9;15(10):1239. doi: 10.3390/ph15101239 (PMC9611342; doi:10.3390/ph15101239)
Supplement: Supplementary file 1 [file pharmaceuticals-15-01239-s001.zip › pharmaceuticals-1845734-supplementary.pdf]

Supplementary Materials: Clinical Efficacy and Safety of Chinese herbal medicine in the Treatment of Uremic Pruritus: A Meta-Analysis of Randomized Controlled Trials

Ping-Hsun Lu, Chien-Cheng Lai, I-Hsin Lin , Fu-Ming Tsai and Po-Hsuan Lu

Figure S1. Forest plot of serum levels in patients with uremic pruritus treated with Chinese herbal medicine: (a) potassium (K), (b) calcium (Ca), (c) phosphorus (P), (d) parathyroid hormone (PTH), and (e) intact parathyroid hormone (iPTH)

Figure S2. Forest plot of indicators of renal function in patients with uremic pruritus treated with Chinese herbal medicine: (a) serum creatinine (SCr), (b) blood urea nitrogen (BUN), (c) estimated glomerular filtration rate (eGFR), and (d) uric acid (UA)

Figure S3. Forest plot of the serum level of inflammation biomarkers in patients with uremic pruritus treated with Chinese herbal medicine: (a) C-reactive protein (CRP), (b) tumor necrosis factor-α (TNF-α), (c) β2-microglobulin (β2-MG), and (d) interleukin-6 (IL-6)

Figure S4. Forest plot of the serum levels in patients with uremic pruritus treated with Chinese herbal medicine: (a) Albumin, (b) Hemoglobin, (c) Aspartate aminotransferase (AST), and (d) Alanine aminotransferase (ALT)

Figure S5. Forest plot of adverse events in patients with uremic pruritus treated with Chinese herbal medicine

Figure S6. Funnel plot of overall effectiveness and visual analogue scale (VAS) ratings for patients with uremic pruritus treated with Chinese herbal medicine

Table S1. Laboratory data for selected studies

Table S2. Components of Chinese herbal medicine in the included studies

Table S3. Grade profile summary of ‘Chinese Herbal Medicine for uremic pruritus’ Quality assessment

Table S4. Search Strategy

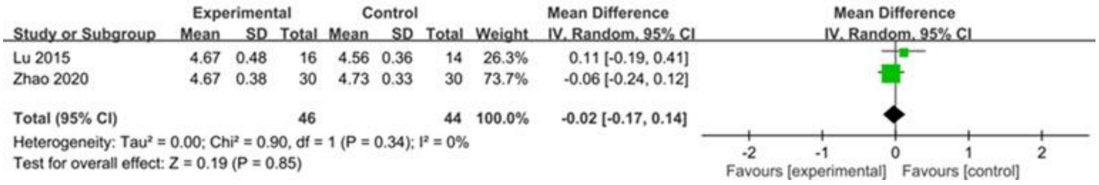

(a) Serum levels of potassium (K) in patients with uremic pruritus treated with Chinese herbal medicine

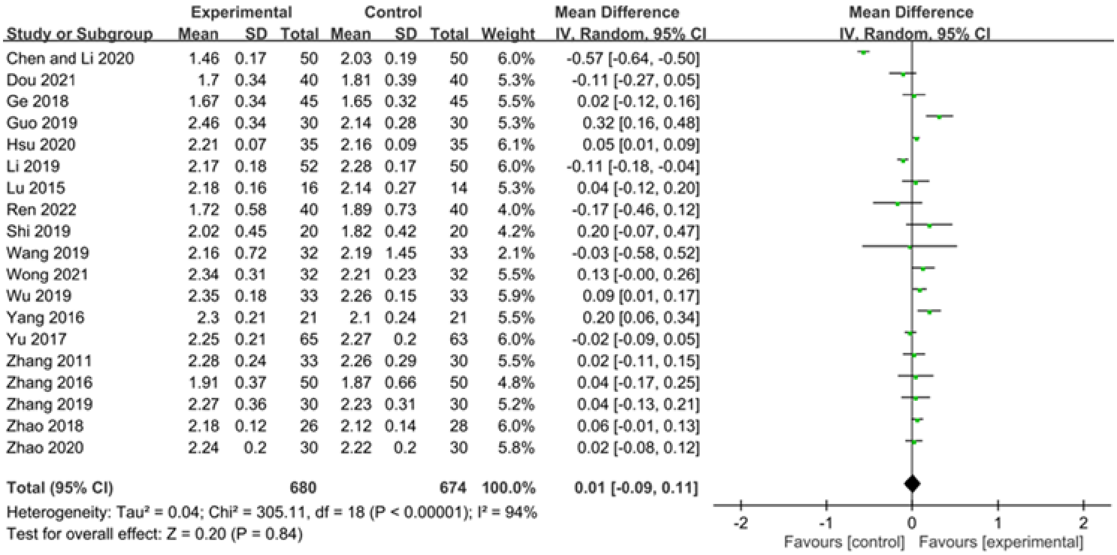

(b) Serum levels of calcium (Ca) in patients with uremic pruritus treated with Chinese herbal medicine

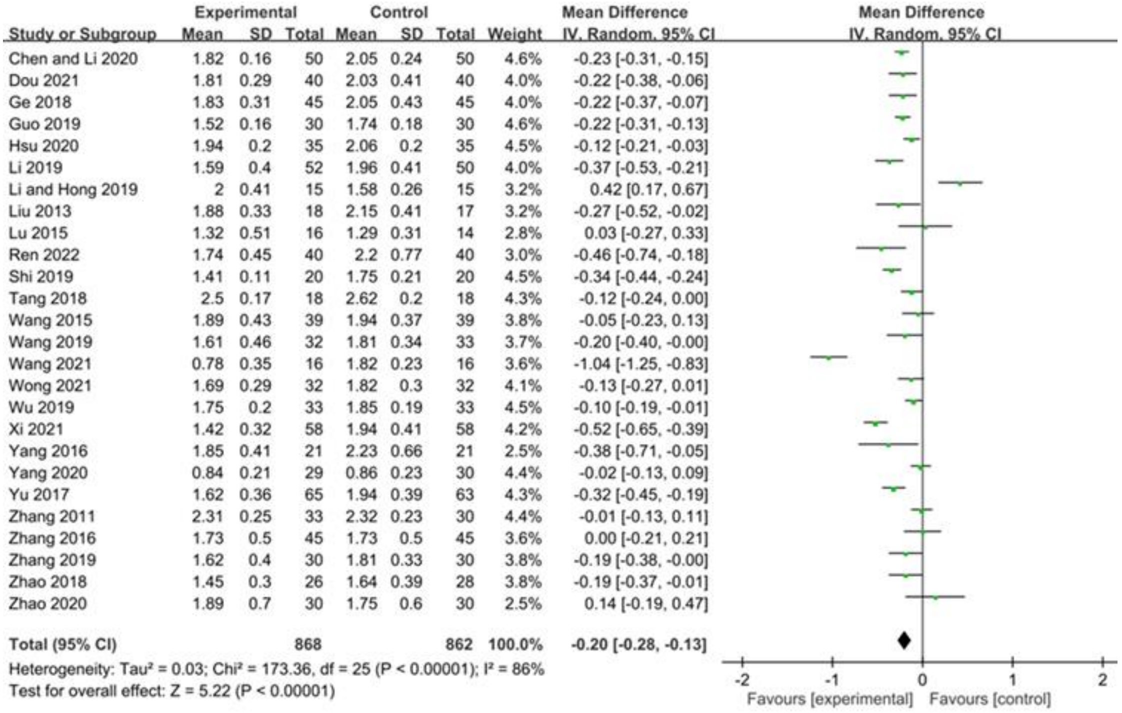

(c) Serum levels of phosphorus (P) in patients with uremic pruritus treated with Chinese herbal medicine

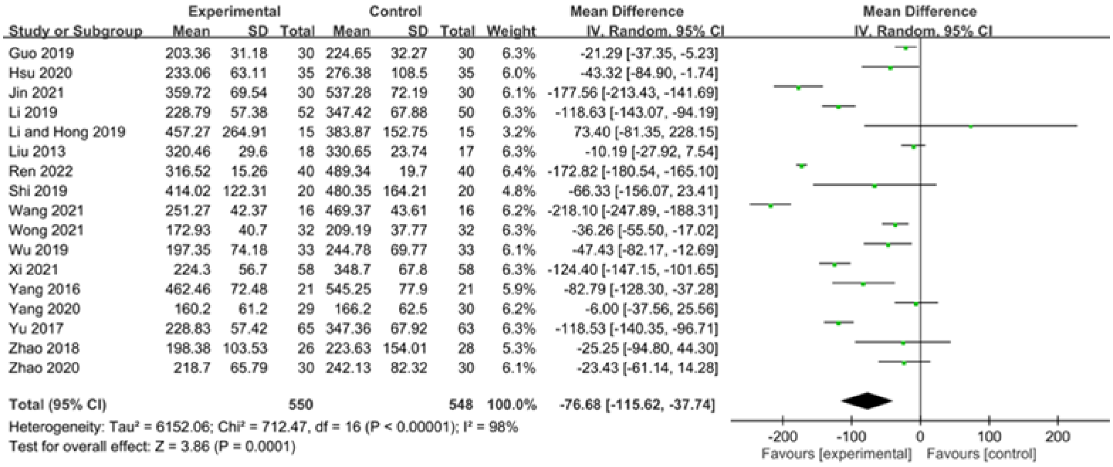

(d) Serum levels of parathyroid hormone (PTH) in patients with uremic pruritus treated with Chinese herbal medicine

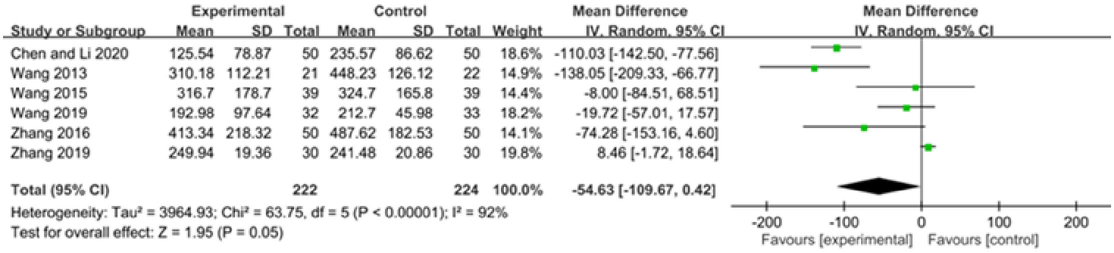

(e) Serum levels of intact parathyroid hormone (iPTH) in patients with uremic pruritus treated with Chinese herbal medicine

Figure S1. Forest plot of serum levels in patients with uremic pruritus treated with Chinese herbal medicine: (a) potassium (K), (b) calcium (Ca), (c) phosphorus (P), (d) parathyroid hormone (PTH), and (e) intact parathyroid hormone (iPTH)

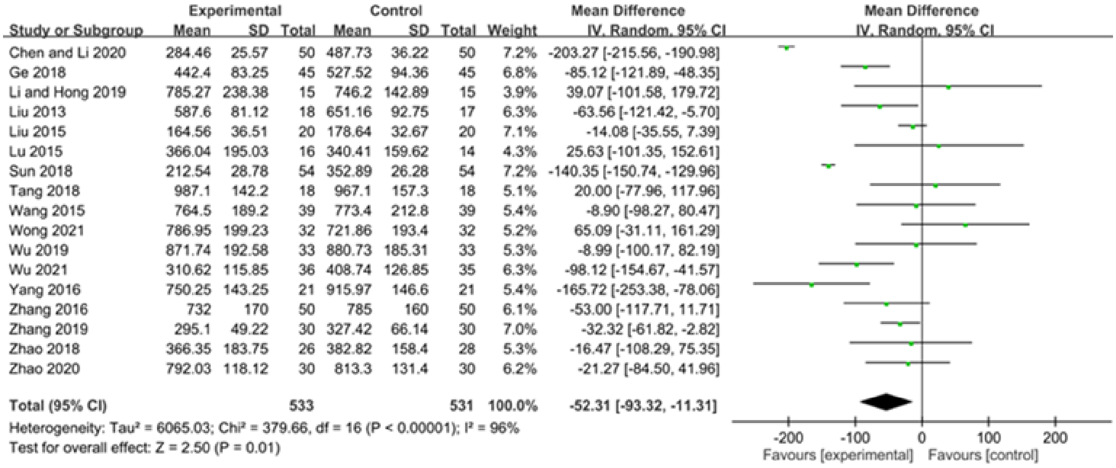

(a) Serum levels of serum creatinine (SCr) in patients with uremic pruritus treated with Chinese herbal medicine

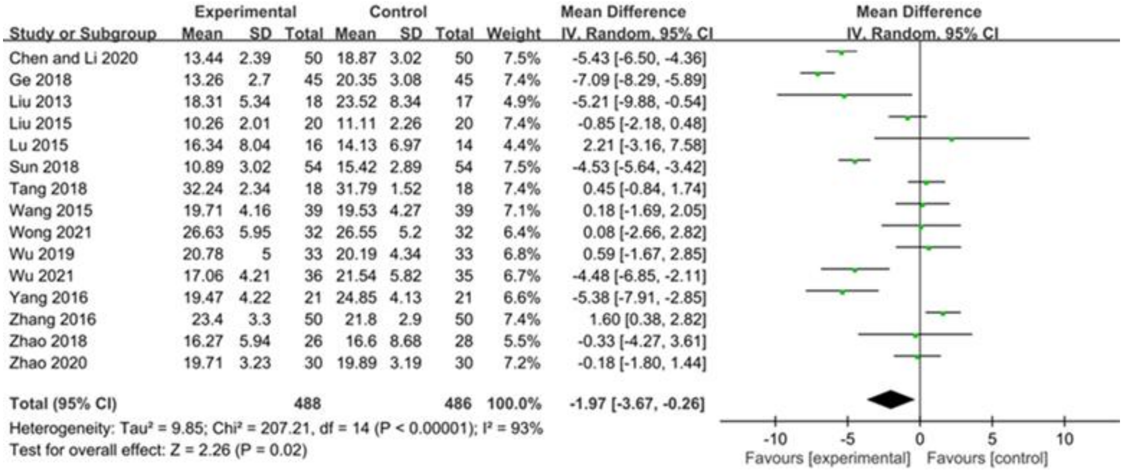

(b) Serum levels of blood urea nitrogen (BUN) in patients with uremic pruritus treated with Chinese herbal medicine

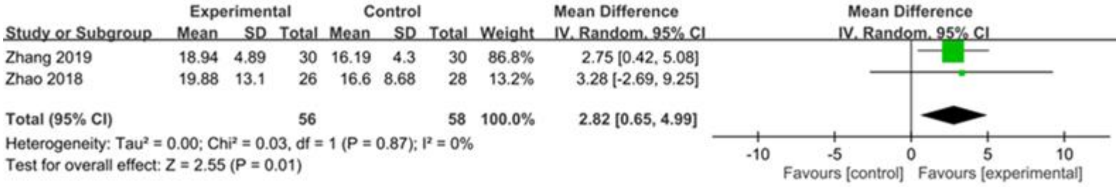

(c) Serum levels of estimated glomerular filtration rate (eGFR) in patients with uremic pruritus treated with Chinese herbal medicine

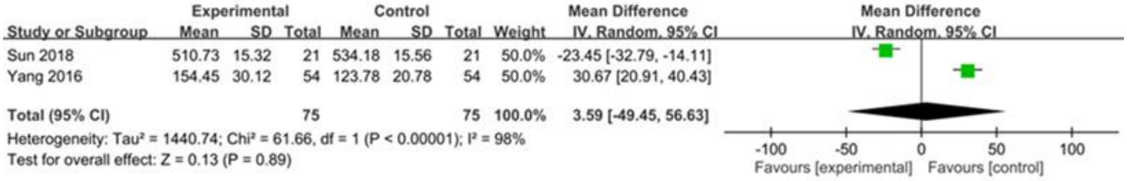

(d) Serum levels of uric acid (UA) in patients with uremic pruritus treated with Chinese herbal medicine

Figure S2. Forest plot of indicators of renal function in patients with uremic pruritus treated with Chinese herbal medicine: (a) serum creatinine (SCr), (b) blood urea nitrogen (BUN), (c) estimated glomerular filtration rate (eGFR), and (d) uric acid (UA)

Study or Subgroup	Experimental Mean	Experimental SD	Experimental Total	Control Mean	Control SD	Control Total	Weight	Mean Difference IV, Random, 95% CI
Cao 2019	2.72	0.81	40	3.17	1.06	40	7.2%	-0.45 [-0.86, -0.04]
Dou 2021	6.11	1.41	40	7.91	2.41	40	6.5%	-1.80 [-2.67, -0.93]
Fan 2020	4.03	3.11	47	2.87	1.64	47	6.2%	1.16 [0.15, 2.17]
Hsu 2020	1.26	1.74	35	2.36	2.39	35	6.3%	-1.10 [-2.08, -0.12]
Li 2019	6.31	4.02	52	8.81	3.38	50	5.3%	-2.50 [-3.94, -1.06]
Sun 2018	8.79	2.12	54	12.45	3.61	54	6.0%	-3.66 [-4.78, -2.54]
Wang 2013	3.05	2.03	21	8.06	1.45	22	6.1%	-5.01 [-6.07, -3.95]
Wang 2019	6.1	1.4	32	7.9	2.4	33	6.3%	-1.80 [-2.75, -0.85]
Wong 2021	3.3	2.15	32	5.27	2.21	32	6.1%	-1.97 [-3.04, -0.90]
Wu 2019	2.46	1.62	33	3.74	2.15	33	6.4%	-1.28 [-2.20, -0.36]
Xi 2021	6.29	1.08	58	8.74	2.16	58	6.9%	-2.45 [-3.07, -1.83]
Yang 2016	12.02	0.92	21	14.22	1.31	21	6.8%	-2.20 [-2.88, -1.52]
Yu 2017	6.27	3.96	65	8.75	3.41	63	5.6%	-2.48 [-3.76, -1.20]
Zhang 2011	4.53	2.75	33	7.18	2.64	30	5.5%	-2.65 [-3.98, -1.32]
Zhang 2016	2.97	1.73	50	4.56	3.91	50	5.8%	-1.59 [-2.78, -0.40]
Zhao 2020	3.43	0.99	30	4.56	1.03	30	7.0%	-1.13 [-1.64, -0.62]
**Total (95% CI)**			**643**			**638**	**100.0%**	**-1.90 [-2.52, -1.27]**

Heterogeneity: Tau² = 1.38; Chi² = 132.61, df = 15 (P < 0.00001); I² = 89%
Test for overall effect: Z = 5.94 (P < 0.00001)

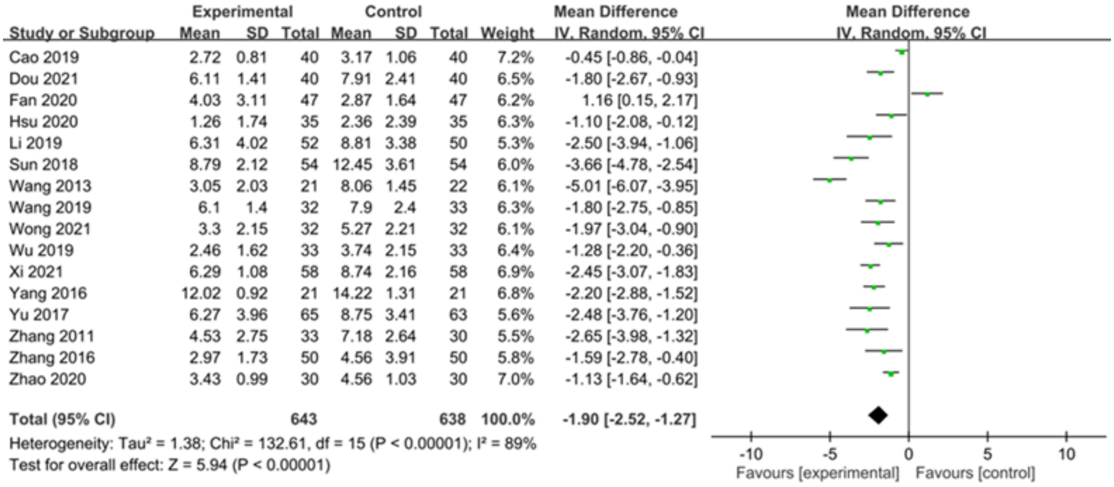

(a) Serum levels of C-reactive protein (CRP) in patients with uremic pruritus treated with Chinese herbal medicine

Study or Subgroup	Experimental Mean	Experimental SD	Experimental Total	Control Mean	Control SD	Control Total	Weight	Mean Difference IV, Random, 95% CI
Dou 2021	37.49	6.12	40	54.28	6.2	40	83.5%	-16.79 [-19.49, -14.09]
Sun 2018	75.12	10.56	54	92.45	20.15	54	16.5%	-17.33 [-23.40, -11.26]
**Total (95% CI)**			**94**			**94**	**100.0%**	**-16.88 [-19.35, -14.41]**

Heterogeneity: Tau² = 0.00; Chi² = 0.03, df = 1 (P = 0.87); I² = 0%
Test for overall effect: Z = 13.41 (P < 0.00001)

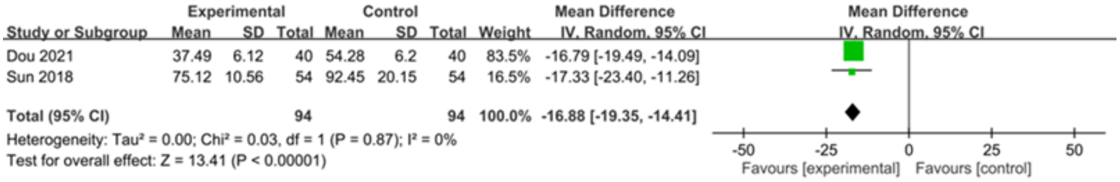

(b) Serum levels of tumor necrosis factor-α (TNF-α) in patients with uremic pruritus treated with Chinese herbal medicine

Study or Subgroup	Experimental Mean	Experimental SD	Experimental Total	Control Mean	Control SD	Control Total	Weight	Mean Difference IV, Random, 95% CI
Chen and Li 2020	6.84	0.69	50	11.54	1.06	50	9.6%	-4.70 [-5.05, -4.35]
Hsu 2020	22.01	2.59	35	23.75	3.45	35	9.1%	-1.74 [-3.17, -0.31]
Jin 2021	11.34	5.41	30	13.72	4.83	30	8.1%	-2.38 [-4.98, 0.22]
Li 2019	22.69	8.87	52	34.68	9.37	50	7.2%	-11.99 [-15.53, -8.45]
Li and Hong 2019	30.15	6.47	15	29.73	8.09	15	5.5%	0.42 [-4.82, 5.66]
Liu 2015	2.14	0.22	20	2.35	0.23	20	9.6%	-0.21 [-0.35, -0.07]
Ren 2022	13.32	4.4	40	19.61	6.9	40	8.2%	-6.29 [-8.83, -3.75]
Wang 2019	2.1	1.4	32	2.6	1.89	33	9.4%	-0.50 [-1.31, 0.31]
Wong 2021	11.16	3.11	32	14.84	2.57	32	9.1%	-3.68 [-5.08, -2.28]
Wu 2019	10.51	2.63	33	15.82	2.56	33	9.2%	-5.31 [-6.56, -4.06]
Xi 2021	21.55	8.24	58	34.74	9.38	58	7.5%	-13.19 [-16.40, -9.98]
Yu 2017	22.75	8.91	65	34.17	9.06	63	7.6%	-11.42 [-14.53, -8.31]
**Total (95% CI)**			**462**			**459**	**100.0%**	**-4.90 [-6.78, -3.02]**

Heterogeneity: Tau² = 9.58; Chi² = 766.14, df = 11 (P < 0.00001); I² = 99%
Test for overall effect: Z = 5.11 (P < 0.00001)

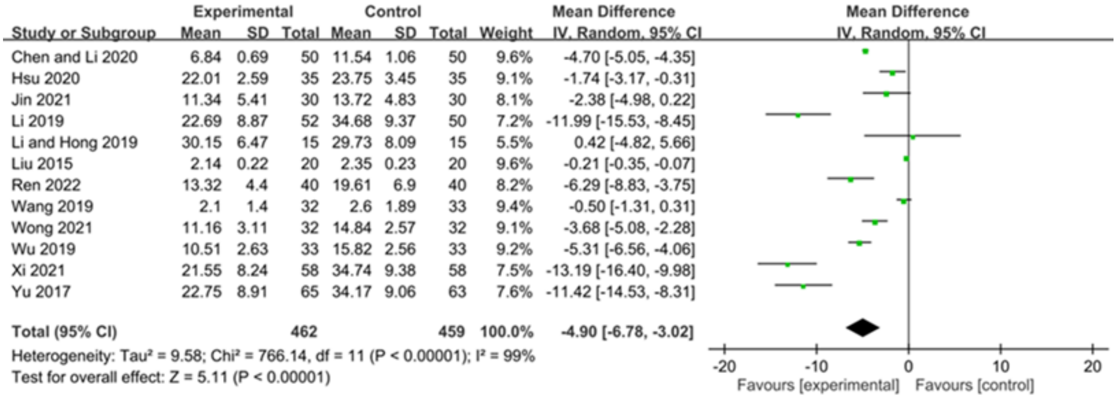

(c) Serum levels of β2-microglobulin (β2-MG) in patients with uremic pruritus treated with Chinese herbal medicine

Study or Subgroup	Experimental Mean	Experimental SD	Experimental Total	Control Mean	Control SD	Control Total	Weight	Mean Difference IV, Random, 95% CI
Dou 2021	23.29	5.78	40	30.11	5.42	40	19.3%	-6.82 [-9.28, -4.36]
Sun 2018	12.56	3.02	54	18.57	4.02	54	24.9%	-6.01 [-7.35, -4.67]
Wong 2021	2.35	1.1	32	3.22	1.24	32	27.7%	-0.87 [-1.44, -0.30]
Wu 2019	2.23	0.58	33	3.31	0.97	33	28.1%	-1.08 [-1.47, -0.69]
**Total (95% CI)**			**159**			**159**	**100.0%**	**-3.36 [-5.26, -1.45]**

Heterogeneity: Tau² = 3.32; Chi² = 70.00, df = 3 (P < 0.00001); I² = 96%
Test for overall effect: Z = 3.46 (P = 0.0005)

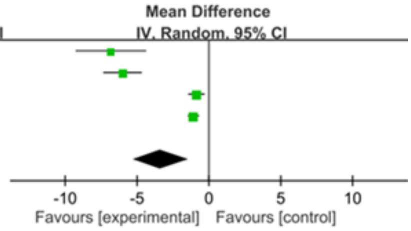

(d) Serum levels of interleukin-6 (IL-6) in patients with uremic pruritus treated with Chinese herbal medicine

Figure S3. Forest plot of the serum level of inflammation biomarkers in patients with uremic pruritus treated with Chinese herbal medicine: (a) C-reactive protein (CRP), (b) tumor necrosis factor-α (TNF-α), (c) β2-microglobulin (β2-MG), and (d) interleukin-6 (IL-6)

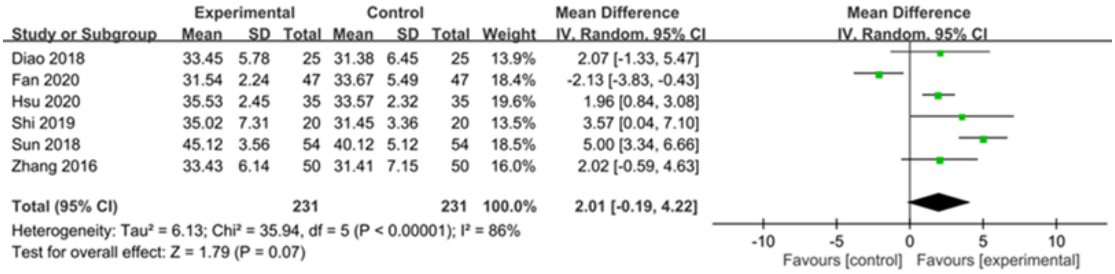

(a) Serum levels of albumin in patients with uremic pruritus treated with Chinese herbal medicine

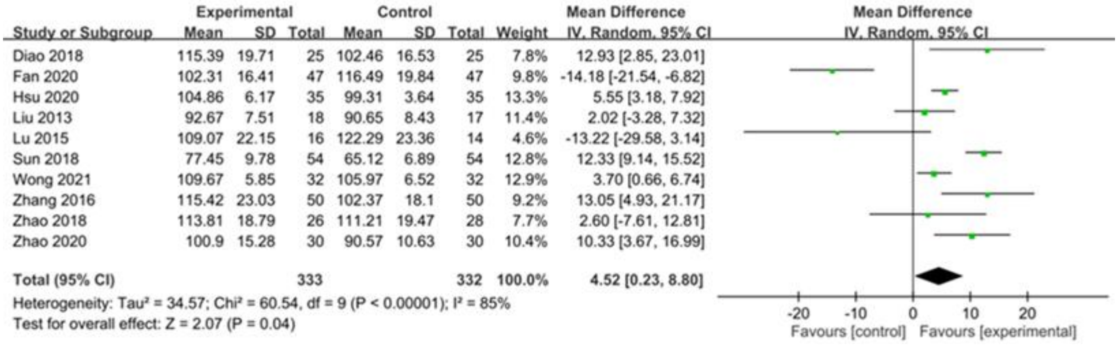

(b) Serum levels of hemoglobin in patients with uremic pruritus treated with Chinese herbal medicine

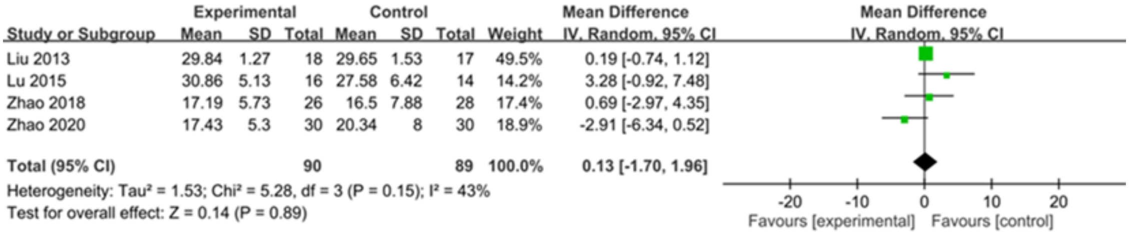

(c) Serum levels of aspartate aminotransferase (AST) in patients with uremic pruritus treated with Chinese herbal medicine

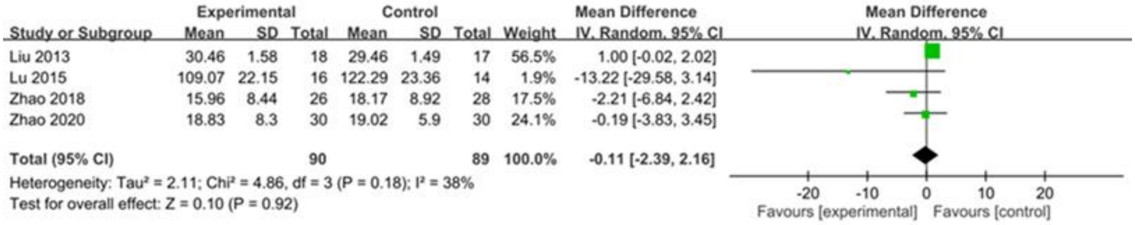

(d) Serum levels of alanine aminotransferase (ALT) in patients with uremic pruritus treated with Chinese herbal medicine

Figure S4. Forest plot of the serum levels in patients with uremic pruritus treated with Chinese herbal medicine: (a) Albumin, (b) Hemoglobin, (c) Aspartate aminotransferase (AST), and (d) Alanine aminotransferase (ALT)

	Experimental		Control			Risk Ratio
Study or Subgroup	Events	Total	Events	Total	Weight	M-H, Random, 95% CI
Chen and Li 2020	6	50	4	50	17.3%	1.50 [0.45, 4.99]
Liu 2015	4	20	7	20	18.3%	0.57 [0.20, 1.65]
Liu 2019	5	51	3	51	16.0%	1.67 [0.42, 6.61]
Ren 2022	2	40	8	40	15.2%	0.25 [0.06, 1.11]
Shi 2019	0	20	4	20	7.9%	0.11 [0.01, 1.94]
Wang 2010	0	28	24	30	8.3%	0.02 [0.00, 0.34]
Wang 2019	1	20	0	20	6.9%	3.00 [0.13, 69.52]
Yang 2020	2	29	1	30	10.1%	2.07 [0.20, 21.60]
**Total (95% CI)**		**258**		**261**	**100.0%**	**0.60 [0.22, 1.63]**
Total events	20		51			

Heterogeneity: $Tau^2 = 1.10$; $Chi^2 = 17.56$, df = 7 (P = 0.01); $I^2 = 60\%$
Test for overall effect: Z = 1.00 (P = 0.32)

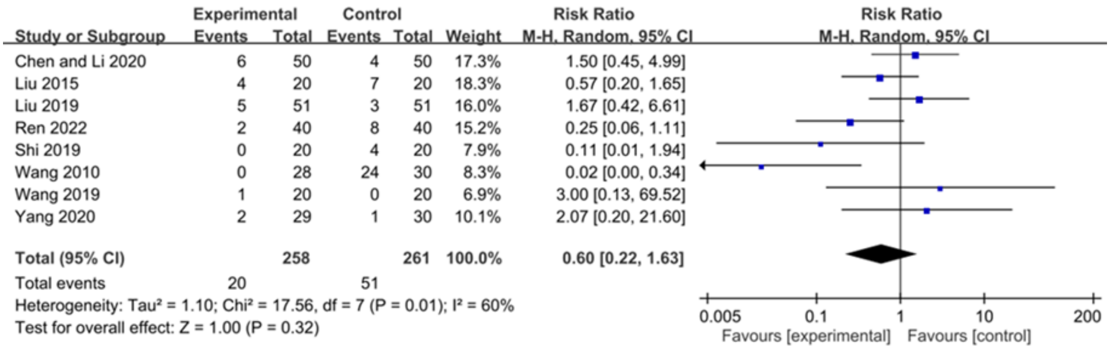

Figure S5. Forest plot of adverse events in patients with uremic pruritus treated with Chinese herbal medicine

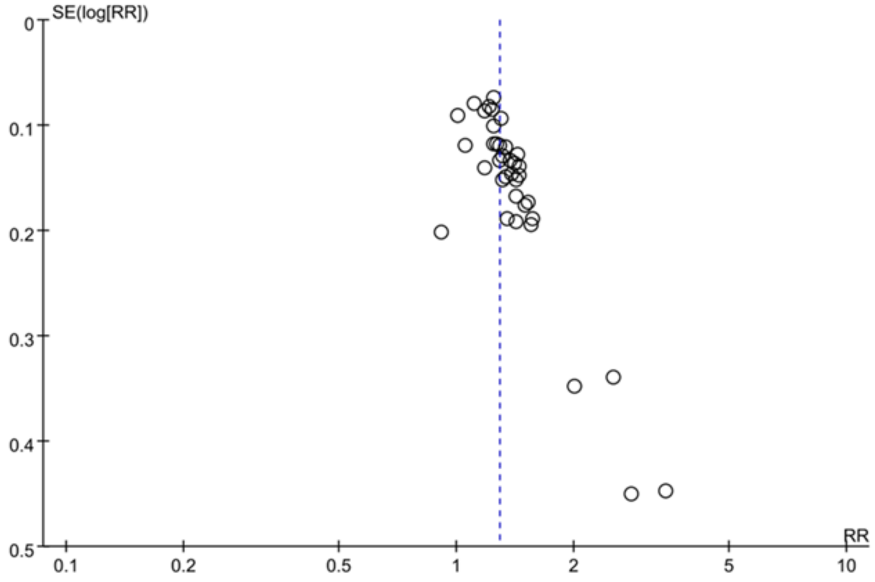

(a) Funnel plot of overall effectiveness for patients with uremic pruritus treated with Chinese herbal medicine

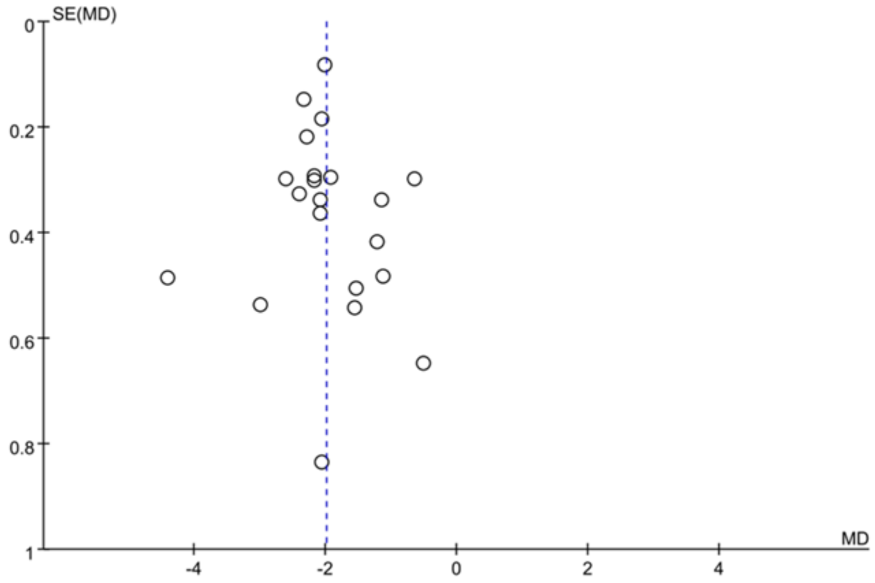

(b) Funnel plot of visual analogue scale (VAS) for patients with uremic pruritus treated with Chinese herbal medicine

Figure S6. Funnel plot of overall effectiveness and visual analogue scale (VAS) ratings for patients with uremic pruritus treated with Chinese herbal medicine

Table S1. Laboratory data for selected studies

Study (year)	Inspection data	Ca (mmol /L)	P (mmol /L)	PTH (ng /L)	iPTH (ng /L)	Scr (μmol /L)	BUN (mmol /L)	UA (μmol /L)	TNF-α (pg /mL)
**Uremic clearance granule (UCG)**									
Yang (2016)	Ca, P, PTH, Scr, BUN, UA, FGF23, CRP	I:2.09(0.24) →2.30(0.21) C:2.09(0.25) →2.10(0.24)	I:2.31(0.48) →1.85(0.41) C:2.17(0.69) →2.23(0.66)	I:562.11(74.42) →462.46(72.48) C:574.22(73.45) →545.25(77.90)	NA	I: 928.25(159.37) →750.25(143.25) C:930.33(165.26) →915.97(146.60)	I:26.26(4.47) →19.47(4.22) C:26.34(4.67) →24.85(4.13)	I: 527.01(15.7) → 510.73(15.32) C:532.23(16.9) → 534.18(15.56)	NA
Sun et al. (2018)	Scr, BUN, UA, hs-CRP, IL-6, TNF-α, Alb, Hb	NA	NA	NA	NA	I:356.12(25.45) →212.54(28.78) C:352.89(26.58) → 289.78(32.56)	I: 20.45(3.56) → 10.89(3.02) C: 20.78(3.69) →15.42(2.89)	I: 128.45(20.12) → 154.45(30.12) C:127.89(23.12) → 123.78(20.78)	I: 115.45(21.45) → 75.12(10.56) C: 115.02(22.02) → 92.45(20.15)
Guo et al. (2019)	Ca, P, PTH, ER	I:1.75(0.26) →2.46(0.34) C:1.72(0.25) →2.14(0.28)	I:1.92(0.27) →1.52(0.16) C:1.91(0.24) →1.74(0.18)	I:241.16(34.48) →203.36(31.18) C:240.18(34.22) →224.65(32.27)	NA	NA	NA	NA	NA
Yu et al. (2017)	Ca, P, PTH, β2-MG, hs-CRP, ER	I: 2.29(0.18) →2.25(0.21) C: 2.32(0.17) →2.27(0.20)	I: 2.34(0.52) →1.62(0.36) C: 2.28(0.45) → 1.94(0.39)	I: 432.35(79.16) →228.83(57.42) C: 428.39(74.7) →347.36(67.92)	NA	NA	NA	NA	NA
Cao (2019)	hs-CRP, ER	NA	NA	NA	NA	NA	NA	NA	NA
Kun et al. (2019)	ER	NA	NA	NA	NA	NA	NA	NA	NA

Li et al. (2019)	Ca, P, PTH, β2-MG, hs-CRP, ER	I: 2.32(0.20) →2.17(0.18)<br>C: 2.30(0.43) →2.28(0.17)	U: 2.41(0.49) →1.59(0.40)<br>C: 2.37(0.43) → 1.96(0.41)	U: 432.41(79.20) →228.79(57.38)<br>C: 428.42(74.66) →347.42(67.88)	NA	NA	NA	NA	NA
Chen and Li et al. (2020)	Ca, P, iPTH, SCr, BUN, β2-MG, ER, ADR	I: 2.89(0.45) →1.46(0.17)<br>C: 2.76(0.52) →2.03(0.19)	I: 2.35(0.34) →1.82(0.16)<br>C: 2.29(0.30) →2.05(0.24)	NA	I: 425.54(105.33) →125.54(78.84)<br>C: 418.87(102.26) →235.57(86.62)	I: 753.58(40.74) →284.46(25.57)<br>C:750.62(42.21) →487.73(36.22)	I:28.41(4.33) →13.44(2.39)<br>C:27.96(4.82) →18.87(3.02)	NA	NA
Xi (2021)	P, PTH, β2-MG, CRP, ER	NA	I:2.38(0.41) →1.42(0.32)<br>C:2.37(0.42) →1.94(0.41)	I:430.8(79.3) →224.3(56.7)<br>C:431.1(79.2) →348.7(67.8)	NA	NA	NA	NA	NA
Li (2021)	ER	NA	NA	NA	NA	NA	NA	NA	NA
**Touxie Jiedu Zhiyang Decoction**									
Wang et al. (2015)	P, iPTH, SCr, BUN, ER	NA	I:2.43(0.82) →1.89(0.43)<br>C:2.38(0.85) →1.94(0.37)	NA	I:459.4(213.2) →316.7(178.7)<br>C:432.7(251.3) →324.7(165.8)	I:826.3(232.6) →764.5(189.2)<br>C:801.5(270.3) →773.4(212.8)	I:22.12(6.67) →19.71(4.16)<br>C:21.86(7.13) →19.53(4.27)	NA	NA
Zhang et al. (2015)	NA	NA	NA	NA	NA	NA	NA	NA	NA
Zhang et al. (2016)	Ca, P, iPTH, Alb, Hb, CRP, HCT, SCr, BUN, AL, ALP	I: 1.76(0.48) →1.91(0.37)<br>C: 1.82(0.54) →1.87(0.66)	I: 1.82(0.54) →1.73(0.50)<br>C: 1.42(0.41) →1.73(0.50)	NA	I:450.46(233.36) →413.34(218.32)<br>C:490.36(187.29) →487.62(182.53)	I:713(180) →732(170)<br>C:778(170) →785(160)	I:21.2(3.8) → 23.4(3.3)<br>C:22.4(2.4) → 21.8(2.9)	NA	NA
Diao et al. (2018)	Alb, Hb, ER, QOL	NA	NA	NA	NA	NA	NA	NA	NA

Shi (2019)	Ca, P, PTH, Alb, Hb, ER, ADR	I: 1.80(0.23) →2.02(0.45)<br>C: 1.81(0.23) →1.82(0.42)	I: 1.87(0.21) →1.41(0.11)<br>C: 1.87(0.21) →1.75(0.21)	I:490.12(175.21) →414.02(122.31)<br>C:490.21(175.25) →480.35(164.21)	NA	NA	NA	NA	NA
Chen (2020)	NA	NA	NA	NA	NA	NA	NA	NA	NA
**Yangxue Runfu Yin**									
Liu (2015)	β2-MG, SCr, BUN, ADR	NA	NA	NA	NA	I:224.56(45.35) →164.56(36.51)<br>C:226.18(41.12) → 178.64(32.67)	I: 16.46(2.31) → 10.26(2.01)<br>C: 17.11(2.28) →11.11(2.26)	NA	NA
Hu (2019)	NA	NA	NA	NA	NA	NA	NA	NA	NA
Wang et al. (2019)	Ca, P, iPTH, β2-MG, CRP, IL-31, QOL, ADR, PSQI	I: 2.15(0.16) →2.16(0.72)<br>C: 2.20(0.72) →2.19(1.45)	I:1.87(0.29) →1.61(0.46)<br>C:1.85(0.37) →1.81(0.34)	NA	I:213.64(89.41) →192.98(97.64)<br>C:214.98(97.64) →212.70(45.98)	NA	NA	NA	NA
Dou (2021)	Ca, P, CRP, TNF-a, IL-6, ER, PSQI	I: 1.62(0.28) →1.70(0.34)<br>C: 1.65(0.34) →1.81(0.39)	I:2.41(0.50) →1.81(0.29)<br>C:2.40(0.53) →2.03(0.41)	NA	NA	NA	NA	NA	I: 63.37(6.19) → 37.49(6.12)<br>C: 62.75(6.26) → 54.28(6.20)
**Other Chinese herbal decoctions**									
Zhu et al. (2004)	ER	NA	NA	NA	NA	NA	NA	NA	NA
He (2006)	ER	NA	NA	NA	NA	NA	NA	NA	NA
Wang et al. (2013)	CRP, iPTH	NA	NA	NA	I:430.12(110.16) →310.18(112.21)<br>C:445.12(122.42)	NA	NA	NA	NA

					→448.23(126.12)				
Ge (2018)	Ca, P, SCr, BUN, ER	I: 1.72(0.36) →1.67(0.34)<br>C: 1.83(0.41) →1.65(0.32)	I:2.42(0.55) →1.83(0.31)<br>C:2.43(0.52) →2.05(0.43)	NA	NA	I:912.72(160.21) →442.40(83.25)<br>C:904.23(174.67) →527.52(94.36)	I:36.25(3.20) →13.26(2.7)<br>C:35.43(3.16) →20.35(3.08)	NA	NA
Tang et al. (2018)	P, SCr, BUN	NA	I:2.65(0.19) →2.50(0.17)<br>C:2.62(0.17) →2.62(0.20)	NA	NA	I:1001(136.1) →987.1(142.2)<br>C:967.1(145.7) →967.1(157.3)	I:32.42(1.89) →32.24(2.34)<br>C:32.76(1.62) →31.79(1.52)	NA	NA
Liu et al. (2019)	QOL, ER, ADR	NA	NA	NA	NA	NA	NA	NA	NA
Fan (2020)	Alb, Hb, CRP, QOL	NA	NA	NA	NA	NA	NA	NA	NA
Yang et al. (2020)	P, PTH, ER, ADR	NA	I:1.83(0.55) →0.84(0.21)<br>C:1.79(0.46) →0.86(0.23)	I:623.8(295.2) →160.2(61.2)<br>C:619.3(289.7) →166.2(62.5)	NA	NA	NA	NA	NA
Zhao (2020)	Ca, P, PTH, SCr, BUN, CRP, Hb, ER, AST, ALT, K, ADR	I:2.24(0.20) →2.24(0.20)<br>C:2.21(0.20) →2.22(0.20)	I:1.90(0.70) →1.89(0.70)<br>C:1.78(0.60) →1.75(0.60)	I:283.97(117.10) →218.70(65.79)<br>C:284.07(122.08) →242.13(82.32)	NA	I:798.47(113.32) →792.03(118.12)<br>C:817.40(135.77) →813.30(131.40)	I:20.28(3.49) →19.71(3.23)<br>C:19.97(2.78) →19.89(3.19)	NA	NA
Jin et al. (2021)	PTH, β2-MG, ER	NA	NA	I:837.31(167.36) →359.72(69.54)<br>C:725.36(187.06) →537.28(72.19)	NA	NA	NA	NA	NA
Wang et al. (2021)	P, PTH, UA	NA	I:2.37(0.38) →0.78(0.35)<br>C:2.32(0.35) →1.82(0.23)	I:667.31(62.53) →251.27(42.37)<br>C: 664.48(61.49) →469.37(43.61)	NA	NA	NA	Wrong unit reported	NA

Wu et al. (2021)	SCr, BUN, ER	NA	NA	NA	NA	I:664.83(165.44)<br>→310.62(115.85)<br>C:665.16(164.83)<br>→408.74(126.85)	I:25.83(6.81)<br>→17.06(4.21)<br>C:26.06(6.92)<br>→21.54(5.82)	NA	NA
Zhou et al. (2021)	PSQI, QOL, ER	NA	NA	NA	NA	NA	NA	NA	NA
**Other Chinese herbal decoctions—Control group with additional treatment**									
Wang et al. (2010)	ER, ADR	NA	NA	NA	NA	NA	NA	NA	NA
Luo et al. (2010)	ER	NA	NA	NA	NA	NA	NA	NA	NA
Zhang et al. (2011)	Ca, P, CRP, ER	I: 2.26(0.27)<br>→2.28(0.24)<br>C: 2.30(0.25)<br>→2.26(0.29)	I: 2.33(0.22)<br>→2.31(0.25)<br>C: 2.30(0.26)<br>→2.32(0.23)	NA	NA	NA	NA	NA	NA
Li (2015)	ER	NA	NA	NA	NA	NA	NA	NA	NA
Wang et al. (2016)	ER	NA	NA	NA	NA	NA	NA	NA	NA
Wu (2016)	ER	NA	NA	NA	NA	NA	NA	NA	NA
Xie et al. (2016)	ER	NA	NA	NA	NA	NA	NA	NA	NA
Li and Hong et al. (2019)	P, PTH, β2-MG, SCr, ER	NA	I: 2.13(0.63)<br>→2.00(0.41)<br>C: 1.89(0.47)<br>→1.58(0.26)	I:552.28(349.46)<br>→457.27(264.91)<br>C:574.32(279.31)<br>→383.87(152.75)	NA	I:835.13(235.93)<br>→785.27(238.38)<br>C:886.93(207.24)<br>→746.2(142.89)	NA	NA	NA
Wu (2019)	Ca, P, PTH, β2-MG, SCr, BUN, hs-CRP,	I: 2.13(0.19)<br>→2.35(0.18)<br>C: 2.19(0.18)<br>→2.26(0.15)	I: 2.13(0.22)<br>→1.75(0.20)<br>C: 2.12(0.21)<br>→1.85(0.19)	I:297.47(105.38)<br>→197.35(74.18)<br>C:292.47(103.59)<br>→244.78(69.77)	NA	I:875.08(190.29)<br>→871.74(192.58)<br>C:885.44(178.29)<br>→880.73(185.31)	I:20.73(5.06)<br>→20.78(5.00)<br>C:20.39(4.36)<br>→20.19(4.34)	NA	NA

	IL-6, ER, ADR								
Hsu (2020)	Ca, P, PTH, β2-MG, hs-CRP, Alb, Hb, ER, ADR	I: 2.12(0.14)<br>→2.21(0.07)<br>C: 2.12(0.13)<br>→2.16(0.09)	I: 2.05(0.21)<br>→1.94(0.20)<br>C: 2.07(0.21)<br>→2.06(0.20)	I:313.74(104.17)<br>→233.06(63.11)<br>C:285.93(116.61)<br>→276.38(108.50)	NA	NA	NA	NA	NA
Li et al. (2020)	NA	NA	NA	NA	NA	NA	NA	NA	NA
Wong (2021)	Ca, P, PTH, SCr, BUN, β2-MG, hs-CRP, IL-6, Hb, ER	I: 2.10(0.21)<br>→2.34(0.31)<br>C: 2.16(0.19)<br>→2.21(0.23)	I:2.06(0.29)<br>→1.69(0.29)<br>C:1.94(0.27)<br>→1.82(0.30)	I:220.61(34.29)<br>→172.93(40.70)<br>C:227.21(33.35)<br>→209.19(37.77)	NA	I:787.99(198.39)<br>→786.95(199.23)<br>C:722.36(193.63)<br>→721.86(193.40)	I:28.25(5.14)<br>→26.63(5.95)<br>C:26.64(4.66)<br>→26.55(5.20)	NA	NA
Ren (2022)	Ca, P, PTH, β2-MG, leptin, SDS, SAS, QOL (SF-36,KDTA), ER, ADR	I: 1.29(0.04)<br>→1.72(0.58)<br>C: 1.23(0.11)<br>→1.89(0.73)	I:2.68(0.92)<br>→1.74(0.45)<br>C:2.63(1.01)<br>→2.20(0.77)	I:546.29(28.31)<br>→316.52(15.26)<br>C:547.42(28.38)<br>→489.34(19.70)	NA	NA	NA	NA	NA
**Patients with uremic pruritus without dialysis**									
Liu (2013)	Ca, P, Mg, PTH, SCr, BUN, Hb,	Wrong unit reported	I:2.21(0.41)<br>→1.88(0.33)<br>C:2.20(0.44)<br>→2.15(0.41)	I:339.33(29.49)<br>→320.46(29.60)<br>C:337.21(22.95)<br>→330.65(23.74)	NA	I:669.16(85.22)<br>→587.60(81.20)<br>C:671.82(92.44)<br>→651.16(92.75)	I:27.33(6.89)<br>→18.31(5.34)<br>C:25.56(8.79)<br>→23.52(8.34)	NA	NA

	WBC,<br>ALT,<br>AST, ER,<br>PT,<br>APTT,<br>PLT								
Lu<br>(2015)	Ca, P,<br>SCr,<br>BUN,<br>UA,<br>eGFR,<br>AST,<br>ALT, K,<br>Hb,<br>urine<br>protein,<br>ER,<br>ADR	I: 2.11(0.18)<br>→2.18(0.16)<br>C: 2.15(0.30)<br>→2.14(0.27)	I:1.28(0.47)<br>→1.32(0.51)<br>C:1.33(0.29)<br>→1.29(0.31)	NA	NA	I:363.81(180.61)<br>→366.04(195.03)<br>C: 330.07(148.53)<br>→340.41(159.62)	I:14.93(7.73)<br>→16.34(8.04)<br>C:13.51(7.32)<br>→14.13(6.97)	Wrong unit reported	NA
Zhao<br>(2018)	Ca, P,<br>PTH,<br>SCr,<br>BUN,<br>eGFR,<br>Hb,<br>ALT,<br>AST, ER,<br>ADR	I: 2.19(0.17)<br>→2.18(0.12)<br>C: 2.12(0.16)<br>→2.12(0.14)	I:1.59(0.42)<br>→1.45(0.30)<br>C:1.66(0.42)<br>→1.64(0.39)	I:213.88(112.80)<br>→198.38(103.53)<br>C:241.24(176.38)<br>→223.63(154.01)	NA	I:413.85(185.55)<br>→366.35(183.75)<br>C:395.79(153.09)<br>→382.82(158.40)	I:20.49(7.21)<br>→16.27(5.94)<br>C:21.54(9.16)<br>→16.60(8.68)	NA	NA
Zhang<br>(2019)	Ca, P,<br>iPTH,<br>SCr,<br>eGFR,<br>ER	I: 2.30(0.40)<br>→2.27(0.36)<br>C: 2.20(0.39)<br>→2.23(0.31)	I:1.89(0.30)<br>→1.62(0.40)<br>C:1.90(0.30)<br>→1.81(0.33)	NA	I:256.94(26.71)<br>→249.94(19.36)<br>C:254.71(26.75)<br>→241.48(20.86)	I:325.03(70.62)<br>→295.10(49.22)<br>C:332.61(79.35)<br>→327.42(66.14)	NA	NA	NA

Table S1. Laboratory data for selected studies (Continued)

Study (year)	FGF23	B2-MG (mg/L)	CRP (mg/L)	Alb (g/L)	Hb (g/L)	IL-6 (pg /mL)	ADR	ALT (U/L)	AST (U/L)
**Uremic clearance granule (UCG)**									
Yang (2016)	I:777.76(120.88) →681.38(115.78) C:779.14(127.78) →797.33(138.36)	NA	I:13.95(1.02) →12.02(0.92) C:14.05(1.11) →14.22(1.31)	NA	NA	NA	NA	NA	NA
Sun et al. (2018)	NA	NA	I:15.78(3.12) →8.79(2.12) C:15.23(3.89) →12.45(3.61)	I:36.45(5.78) →45.12(3.56) C:36.28(5.45) →40.12(5.12)	I:56.89(10.23) →77.45(9.78) C:56.80(9.45) →65.12(6.89)	I: 23.89(5.02) →12.56(3.02) C:23.78(4.25) →18.57(4.02)	NA	NA	NA
Guo et al. (2019)	NA	NA	NA	NA	NA	NA	NA	NA	NA
Yu et al. (2017)	NA	I:48.57(12.21) →22.75(8.91) C:50.49(13.46) →34.17(9.06)	I:12.73(3.19) →6.27(3.96) C:13.09(3.26) →8.75(3.41)	NA	NA	NA	NA	NA	NA
Cao (2019)	NA	NA	I:3.16(1.15) →2.72(0.81) C:3.15(1.12) →3.17(1.06)	NA	NA	NA	NA	NA	NA
Kun et al. (2019)	NA	NA	NA	NA	NA	NA	NA	NA	NA
Li et al. (2019)	NA	I:52.67(12.18) →22.69(8.87) C:50.52(13.51) →34.68(9.37)	I:12.69(3.21) →6.31(4.02) C:13.12(3.31) → 8.81(3.38)	NA	NA	NA	NA	NA	NA
Chen and Li et al. (2020)	NA	I:14.01(0.97) →6.84(0.69) C:13.87(0.82) →11.54(1.06)	NA	NA	NA	NA	I: 6/50 C: 4/50	NA	NA
Xi (2021)	NA	I:52.57(12.79) →21.55(8.24) C:52.68(12.81) →34.74(9.38)	I:12.98(3.14) →6.29(1.08) C:12.97(3.12) →8.74(2.16)	NA	NA	NA	NA	NA	NA

Li (2021)	NA	NA	NA	NA	NA	NA	NA	NA	NA
**Touxie Jiedu Zhiyang Decoction**									
Wang et al. (2015)	NA	NA	NA	NA	NA	NA	NA	NA	NA
Zhang et al. (2015)	NA	NA	NA	NA	NA	NA	NA	NA	NA
Zhang et al. (2016)	NA	NA	I:4.39(4.15) →2.97(1.73) C:4.27(4.23) →4.56(3.91)	I:31.21(4.54) →33.43(6.14) C:30.03(4.12) →31.41(7.15)	I:102.12(15.21) →115.42(23.03) C:96.41(23.73) →102.37(18.10)	NA	NA	NA	NA
Diao et al. (2018)	NA	NA	NA	I:30.67(3.98) →33.45(5.78) C:30.48(4.09) →31.38(6.45)	I:97.25(20.03) →115.39(19.71) C:96.38(19.87) →102.46(16.53)	NA	NA	NA	NA
Shi (2019)	NA	NA	NA	I:30.12(2.21) →35.02(7.31) C:30.12(2.21) →31.45(3.36)	Incorrect unit conversion	NA	I: 0/20 C: 4/20	NA	NA
Chen (2020)	NA	NA	NA	NA	NA	NA	NA	NA	NA
**Yangxue Runfu Yin**									
Liu (2015)	NA	I:2.78(0.21) →2.14(0.22) C:2.81(0.22) →2.35(0.23)	NA	NA	NA	NA	I: 4/20 C: 7/20	NA	NA
Hu (2019)	NA	NA	NA	NA	NA	NA	NA	NA	NA
Wang et al. (2019)	NA	I:2.80(2.40) →2.10(1.40) C:2.70(1.20) →2.60(1.89)	I:7.90(2.60) → 6.10(1.40) C:8.00(2.70) → 7.90(2.40)	NA	NA	NA	I: 1/20 C: 0/20	NA	NA
Dou (2021)	NA	NA	I:7.91(2.61) → 6.11(1.41) C:8.01(2.71) → 7.91(2.41)	NA	NA	I: 32.48(6.06) →23.29(5.78) C:31.72(5.92) →30.11(5.42)	NA	NA	NA

Other Chinese herbal decoctions

Zhu et al. (2004)	NA	NA	NA	NA	NA	NA	NA	NA	NA
He (2006)	NA	NA	NA	NA	NA	NA	NA	NA	NA
Wang et al. (2013)	NA	NA	I:7.59(4.22) → 3.05(2.03) C:7.89(4.32) →8.06(1.45)	NA	NA	NA	NA	NA	NA
Ge (2018)	NA	NA	NA	NA	NA	NA	NA	NA	NA
Tang et al. (2018)	NA	NA	NA	NA	NA	NA	NA	NA	NA
Liu et al. (2019)	NA	NA	NA	NA	NA	NA	I: 5/51 C: 3/51	NA	NA
Fan (2020)	NA	NA	I:4.37(4.13) → 4.03(3.11) C:4.29(4.38) →2.87(1.64)	I:30.62(4.11) →31.54(2.24) C:30.49(4.21) →33.67(5.49)	I:96.48(19.28) →102.31(16.41) C:96.67(19.35) →116.49(19.84)	NA	NA	NA	NA
Yang et al. (2020)	NA	NA	NA	NA	NA	NA	I: 2/29 C: 1/30	NA	NA
Zhao (2020)	NA	NA	I:7.85(2.42) → 3.43(0.99) C:7.98(2.52) →4.56(1.03)	NA	I:90.59(16.8) →100.9(15.28) C:91.2(11.96) →90.57(10.63)	NA	I: 0/30 C: 0/30	I:16.23(5.9) →18.83(8.3) C:19.79(8.0) →19.02(5.9)	I:19.15(7.9) →17.43(5.3) C:19.91(9.6) →20.34(8.0)
Jin et al. (2021)	NA	I:17.63(6.28) →11.34(5.41) C:16.84(5.76) →13.72(4.83)	NA	NA	NA	NA	NA	NA	NA
Wang et al. (2021)	NA	NA	NA	NA	NA	NA	NA	NA	NA

Wu et al. (2021)	NA	NA	NA	NA	NA	NA	NA	NA	NA
Zhou et al. (2021)	NA	NA	NA	NA	NA	NA	NA	NA	NA
**Other Chinese herbal decoctions—Control group with additional treatment**									
Wang et al. (2010)	NA	NA	NA	NA	NA	NA	I: 0/28 C: 24/30	NA	NA
Luo et al. (2010)	NA	NA	NA	NA	NA	NA	NA	NA	NA
Zhang et al. (2011)	NA	NA	I:7.27(2.46) → 4.53(2.75) C:7.36(2.35) →7.18(2.64)	NA	NA	NA	NA	NA	NA
Li (2015)	NA	NA	NA	NA	NA	NA	NA	NA	NA
Wang et al. (2016)	NA	NA	NA	NA	NA	NA	NA	NA	NA
Wu (2016)	NA	NA	NA	NA	NA	NA	NA	NA	NA
Xie (2016)	NA	NA	NA	NA	NA	NA	NA	NA	NA
Li and Hong et al. (2019)	NA	I:32.62(8.01) →30.15(6.47) C:37.37(10.37) →29.73(8.09)	NA	NA	NA	NA	NA	NA	NA
Wu (2019)	NA	I:16.77(2.29) →10.51(2.63) C:16.70(2.42) →15.82(2.56)	I:7.22(2.29) → 2.46(1.62) C:7.18(2.28) →3.74(2.15)	NA	NA	I:4.88(1.76) →2.23(0.58) C:4.93(1.88) →3.31(0.97)	I: 0/33 C: 0/33	NA	NA
Hsu (2020)	NA	I:23.91(3.16) →22.01(2.59) C:24.06(3.40) →23.75(3.45)	I:2.50(2.75) → 1.26(1.74) C:2.61(2.85) →2.36(2.39)	I:33.26(2.94) →35.53(2.45) C:33.75(2.93) →33.57(2.32)	I:100.20(5.00) →104.86(6.17) C:99.83(5.39) →99.31(3.64)	NA	I: 0/35 C: 0/35	NA	NA

Li et al. (2020)	NA	NA	NA	NA	NA	NA	NA	NA	NA
Wong (2021)	NA	I:16.35(2.18) →11.16(3.11) C:15.78(2.51) →14.84(2.57)	I:7.45(2.64) → 3.30(2.15) C:7.42(2.35) →5.27(2.21)	NA	I:99.61(4.61) →109.67(5.85) C:99.19(5.85) →105.97(6.52)	I:4.98(1.64) →2.35(1.10) C:4.91(1.69) →3.22(1.24)	NA	NA	NA
Ren (2022)	NA	I:22.94(8.40) →13.32(4.40) C:22.74(8.27) →19.61(6.90)	NA	NA	NA	NA	I: 2/40 C: 8/40	NA	NA
**Patients with uremic pruritus without dialysis**									
Liu (2013)	NA	NA	NA	NA	I:82.72(9.54) →92.67(7.51) C:82.06(10.35) →90.65(8.43)	NA	NA	I:28.84(1.46) →30.46(1.58) C:27.87(1.65) →29.46(1.49)	I:29.42(1.35) →29.84(1.27) C:29.01(1.66) →29.65(1.53)
Lu (2015)	NA	NA	NA	NA	I:109.21(20.45) →109.07(22.15) C:120.00(24.24) →122.29(23.36)	NA	I: 0/14 C: 0/16	I:109.21(20.45) → 109.07(22.15) C:120.00(24.24) → 122.29(23.36)	I:28.21(7.02) →30.86(5.13) C:28.06(6.94) →27.58(6.42)
Zhao (2018)	NA	NA	NA	NA	I:106.38(18.79) →113.81(18.79) C:106.04(24.73) →111.21(19.47)	NA	I: 0/28 C: 0/30	(mmol/L) I:15.54(8.97) →15.96(8.44) C: 17.39(8.60) →18.17(8.92)	(mmol/L) I:17.38(5.43) →17.19(5.73) C: 17.04(6.74) →16.50(7.88)
Zhang (2019)	NA	NA	NA	NA	NA	NA	NA	NA	NA

Table S1. Laboratory data for selected studies (Continued)

Study (year)	eGFR (mL/min/1.73m²)	K (mmol/L)	Mg (mmol/L)	IL-31 (pg/ml)	PT (S)	APTT (S)	PLT (10^9/L)	WBC (10^9/L)	Urine protein (mg/24hr)
**Uremic clearance granule (UCG)**									
Yang (2016)	NA	NA	NA	NA	NA	NA	NA	NA	NA
Sun et al. (2018)	NA	NA	NA	NA	NA	NA	NA	NA	NA
Guo et al. (2019)	NA	NA	NA	NA	NA	NA	NA	NA	NA
Yu et al. (2017)	NA	NA	NA	NA	NA	NA	NA	NA	NA
Cao (2019)	NA	NA	NA	NA	NA	NA	NA	NA	NA
Kun et al. (2019)	NA	NA	NA	NA	NA	NA	NA	NA	NA
Li et al. (2019)	NA	NA	NA	NA	NA	NA	NA	NA	NA
Chen and Li et al. (2020)	NA	NA	NA	NA	NA	NA	NA	NA	NA
Xi (2021)	NA	NA	NA	NA	NA	NA	NA	NA	NA
Li (2021)	NA	NA	NA	NA	NA	NA	NA	NA	NA
**Touxie Jiedu Zhiyang Decoction**									
Wang et al. (2015)	NA	NA	NA	NA	NA	NA	NA	NA	NA

Zhang et al. (2015)	NA	NA	NA	NA	NA	NA	NA	NA	NA
Zhang et al. (2016)	NA	NA	NA	NA	NA	NA	NA	NA	NA
Diao et al. (2018)	NA	NA	NA	NA	NA	NA	NA	NA	NA
Shi (2019)	NA	NA	NA	NA	NA	NA	NA	NA	NA
Chen (2020)	NA	NA	NA	NA	NA	NA	NA	NA	NA
**Yangxue Runfu Yin**									
Liu (2015)	NA	NA	NA	NA	NA	NA	NA	NA	NA
Hu (2019)	NA	NA	NA	NA	NA	NA	NA	NA	NA
Wang et al. (2019)	NA	NA	NA	I:32.10(11.20) → 23.70(12.80) C:31.40(9.78) → 30.12(10.60)	NA	NA	NA	NA	NA
Dou (2021)	NA	NA	NA	NA	NA	NA	NA	NA	NA
**Other Chinese herbal decoctions**									
Zhu et al. (2004)	NA	NA	NA	NA	NA	NA	NA	NA	NA
He (2006)	NA	NA	NA	NA	NA	NA	NA	NA	NA
Wang et al. (2013)	NA	NA	NA	NA	NA	NA	NA	NA	NA

Ge (2018)	NA	NA	NA	NA	NA	NA	NA	NA	NA
Tang et al. (2018)	NA	NA	NA	NA	NA	NA	NA	NA	NA
Liu et al. (2019)	NA	NA	NA	NA	NA	NA	NA	NA	NA
Fan (2020)	NA	NA	NA	NA	NA	NA	NA	NA	NA
Yang et al. (2020)	NA	NA	NA	NA	NA	NA	NA	NA	NA
Zhao (2020)	NA	I:4.81(0.45) →4.67(0.38) C:4.70(0.43) →4.73(0.33)	NA	NA	NA	NA	NA	NA	NA
Jin et al. (2021)	NA	NA	NA	NA	NA	NA	NA	NA	NA
Wang et al. (2021)	NA	NA	NA	NA	NA	NA	NA	NA	NA
Wu et al. (2021)	NA	NA	NA	NA	NA	NA	NA	NA	NA
Zhou et al. (2021)	NA	NA	NA	NA	NA	NA	NA	NA	NA
**Other Chinese herbal decoctions—Control group with additional treatment**									
Wang et al. (2010)	NA	NA	NA	NA	NA	NA	NA	NA	NA
Luo et al. (2010)	NA	NA	NA	NA	NA	NA	NA	NA	NA

Zhang et al. (2011)	NA	NA	NA	NA	NA	NA	NA	NA	NA
Li (2015)	NA	NA	NA	NA	NA	NA	NA	NA	NA
Wang et al. (2016)	NA	NA	NA	NA	NA	NA	NA	NA	NA
Wu (2016)	NA	NA	NA	NA	NA	NA	NA	NA	NA
Xie (2016)	NA	NA	NA	NA	NA	NA	NA	NA	NA
Li and Hong et al. (2019)	NA	NA	NA	NA	NA	NA	NA	NA	NA
Wu (2019)	NA	NA	NA	NA	NA	NA	NA	NA	NA
Hsu (2020)	NA	NA	NA	NA	NA	NA	NA	NA	NA
Li et al. (2020)	NA	NA	NA	NA	NA	NA	NA	NA	NA
Wong (2021)	NA	NA	NA	NA	NA	NA	NA	NA	NA
Ren (2022)	NA	NA	NA	NA	NA	NA	NA	NA	NA
**Patients with uremic pruritus without dialysis**									
Liu (2013)	NA	NA	I:1.48(0.32) →0.95(0.24) C:1.47(0.33) →1.10(0.25)	NA	I:12.66(1.73) →14.68(1.36) C:12.79(1.93) →13.39(1.67)	I:32.27(7.25) →35.48(5.99) C:31.86(6.52) →31.23(5.97)	I:194.83(28.49) →178.44(21.98) C:196.71(22.98) →193.00(19.93)	I:5.36(1.25) →5.59(0.95) C:5.47(1.15) →5.55(0.83)	NA
Lu (2015)	Incorrect data reported	I:4.72(0.67) →4.67(0.48) C:4.51(0.52) →4.56(0.36)	NA	NA	NA	NA	NA	NA	I:1398.10(857.96) → 1474.36(753.72) C:1609.55(1177.88)

									→ 1784.44(984.72)
Zhao (2018)	I:15.48(8.25) → 19.88(13.10) C:15.39(7.29) →16.60(8.68)	NA	NA	NA	NA	NA	NA	NA	NA
Zhang (2019)	I:16.44(4.13) →18.94(4.89) C:15.49(3.56) →16.19(4.30)	NA	NA	NA	NA	NA	NA	NA	NA

Table S2. Components of Chinese herbal medicine in the included studies

References	Chinese herbal medicine	Components
Yang (2016)	Uremic clearance granule	Rheum officinale (Dahuang), Bupleurum chinens (Chaihu), Astragalus membranaceus (Huangqi), Morus alba (Sangbaipi), Codonopsis pilosula (Dangshen), Paeonia lactiflora (Baishao), Ligusticum chuanxiong (Chuanxiong), Sophora flavescens (Kushen), Chrysanthemum morifolium (Juhua), Pinellia ternata (Banxia), Atractylodes macrocephala (Baizhu), Poria cocos (Fuling), Polygonum multiflorum (Heshouwu), Salviae Miltiorrhizae (Danshen), Herba Plantaginis (Cheqiancao), Glycyrrhiza uralensis (Gancao)
Sun et al. (2018)	Uremic clearance granule	Rheum officinale (Dahuang), Bupleurum chinens (Chaihu), Astragalus membranaceus (Huangqi), Morus alba (Sangbaipi), Codonopsis pilosula (Dangshen), Paeonia lactiflora (Baishao), Ligusticum chuanxiong (Chuanxiong), Sophora flavescens (Kushen), Chrysanthemum morifolium (Juhua), Pinellia ternata (Banxia), Atractylodes macrocephala (Baizhu), Poria cocos (Fuling), Polygonum multiflorum (Heshouwu), Salviae Miltiorrhizae (Danshen), Herba Plantaginis (Cheqiancao), Glycyrrhiza uralensis (Gancao)
Guo et al. (2019)	Uremic clearance granule	Rheum officinale (Dahuang), Bupleurum chinens (Chaihu), Astragalus membranaceus (Huangqi), Morus alba (Sangbaipi), Codonopsis pilosula (Dangshen), Paeonia lactiflora (Baishao), Ligusticum chuanxiong (Chuanxiong), Sophora flavescens (Kushen), Chrysanthemum morifolium (Juhua), Pinellia ternata (Banxia), Atractylodes macrocephala (Baizhu), Poria cocos (Fuling), Polygonum multiflorum (Heshouwu), Salviae Miltiorrhizae (Danshen), Herba Plantaginis (Cheqiancao), Glycyrrhiza uralensis (Gancao)
Yu et al. (2017)	Uremic clearance granule	Rheum officinale (Dahuang), Bupleurum chinens (Chaihu), Astragalus membranaceus (Huangqi), Morus alba (Sangbaipi), Codonopsis pilosula (Dangshen), Paeonia lactiflora (Baishao), Ligusticum chuanxiong (Chuanxiong), Sophora flavescens (Kushen), Chrysanthemum morifolium (Juhua), Pinellia ternata (Banxia), Atractylodes macrocephala (Baizhu), Poria cocos (Fuling), Polygonum multiflorum (Heshouwu), Salviae Miltiorrhizae (Danshen), Herba Plantaginis (Cheqiancao), Glycyrrhiza uralensis (Gancao)
Cao (2019)	Uremic clearance granule	Rheum officinale (Dahuang), Bupleurum chinens (Chaihu), Astragalus membranaceus (Huangqi), Morus alba (Sangbaipi), Codonopsis pilosula (Dangshen), Paeonia lactiflora (Baishao), Ligusticum chuanxiong (Chuanxiong), Sophora flavescens (Kushen), Chrysanthemum morifolium (Juhua), Pinellia ternata (Banxia), Atractylodes macrocephala (Baizhu), Poria cocos (Fuling), Polygonum multiflorum (Heshouwu), Salviae Miltiorrhizae (Danshen), Herba Plantaginis (Cheqiancao), Glycyrrhiza uralensis (Gancao)
Kun et al.(2019)	Uremic clearance granule	Rheum officinale (Dahuang), Bupleurum chinens (Chaihu), Astragalus membranaceus (Huangqi), Morus alba (Sangbaipi), Codonopsis pilosula (Dangshen), Paeonia lactiflora (Baishao), Ligusticum chuanxiong (Chuanxiong), Sophora flavescens (Kushen), Chrysanthemum morifolium (Juhua), Pinellia ternata (Banxia), Atractylodes macrocephala (Baizhu), Poria cocos (Fuling), Polygonum multiflorum (Heshouwu), Salviae Miltiorrhizae (Danshen), Herba Plantaginis (Cheqiancao), Glycyrrhiza uralensis (Gancao)
Li et al. (2019)	Uremic clearance granule	Rheum officinale (Dahuang), Bupleurum chinens (Chaihu), Astragalus membranaceus (Huangqi), Morus alba (Sangbaipi), Codonopsis pilosula (Dangshen), Paeonia lactiflora (Baishao), Ligusticum chuanxiong (Chuanxiong), Sophora flavescens (Kushen), Chrysanthemum morifolium (Juhua), Pinellia ternata (Banxia), Atractylodes macrocephala (Baizhu), Poria cocos (Fuling), Polygonum multiflorum (Heshouwu), Salviae Miltiorrhizae (Danshen), Herba Plantaginis (Cheqiancao), Glycyrrhiza uralensis (Gancao)
Chen and Li et al. (2020)	Uremic clearance granule	Rheum officinale (Dahuang), Bupleurum chinens (Chaihu), Astragalus membranaceus (Huangqi), Morus alba (Sangbaipi), Codonopsis pilosula (Dangshen), Paeonia lactiflora (Baishao), Ligusticum chuanxiong (Chuanxiong), Sophora flavescens (Kushen), Chrysanthemum morifolium (Juhua), Pinellia ternata (Banxia), Atractylodes macrocephala (Baizhu), Poria cocos (Fuling), Polygonum multiflorum (Heshouwu), Salviae Miltiorrhizae (Danshen), Herba Plantaginis (Cheqiancao), Glycyrrhiza uralensis (Gancao)
Xi (2021)	Uremic clearance granule	Rheum officinale (Dahuang), Bupleurum chinens (Chaihu), Astragalus membranaceus (Huangqi), Morus alba (Sangbaipi), Codonopsis pilosula (Dangshen), Paeonia lactiflora (Baishao), Ligusticum chuanxiong (Chuanxiong), Sophora flavescens (Kushen), Chrysanthemum morifolium (Juhua), Pinellia ternata (Banxia), Atractylodes macrocephala (Baizhu), Poria cocos (Fuling), Polygonum multiflorum (Heshouwu), Salviae Miltiorrhizae (Danshen), Herba Plantaginis (Cheqiancao), Glycyrrhiza uralensis (Gancao)

Li (2021)	Uremic clearance granule	Rheum officinale (Dahuang), Bupleurum chinens (Chaihu), Astragalus membranaceus (Huangqi), Morus alba (Sangbaipi), Codonopsis pilosula (Dangshen), Paeonia lactiflora (Baishao), Ligusticum chuanxiong (Chuanxiong), Sophora flavescens (Kushen), Chrysanthemum morifolium (Juhua), Pinellia ternata (Banxia), Atractylodes macrocephala (Baizhu), Poria cocos (Fuling), Polygonum multiflorum (Heshouwu), Salviae Miltiorrhizae (Danshen), Herba Plantaginis (Cheqiancao), Glycyrrhiza uralensis (Gancao)
Wang et al. (2015)	Touxie Jiedu Zhiyang Decoction	Astragalus membranaceus (Huangqi), Scutellaria baicalnsis (Huangqin), Rehmannia glutinosa (Dihuang), Paeonia lactiflora (Baishao), Angelicae Sinensis (Danggui), Dipsaci Radix (Xuduan), Persicae Semen (Taoren), Eucommia ulmoides (Duzhong), Codonopsis pilosula (Dangshen), Chaenomelis Fructus (Mugua), Carthami Flos (Honghua), Spatholobi Caulis (Jixueteng), Clematidis Radix (Weilingxian), Angelicae Dahuricae Radix (Baizhi), Achyranthis Bidentatae Radix (Niuxi), Rheum officinale (Dahuang), Glycyrrhiza uralensis (Gancao)
Zhang et al. (2015)	Touxie Zhiyang Decoction	Astragalus membranaceus (Huangqi), Scutellaria baicalnsis (Huangqin), Rehmannia glutinosa (Dihuang), Paeonia lactiflora (Baishao), Angelicae Sinensis (Danggui), Dipsaci Radix (Xuduan), Persicae Semen (Taoren), Eucommia ulmoides (Duzhong), Codonopsis pilosula (Dangshen), Chaenomelis Fructus (Mugua), Carthami Flos (Honghua), Spatholobi Caulis (Jixueteng), Clematidis Radix (Weilingxian), Angelicae Dahuricae Radix (Baizhi), Achyranthis Bidentatae Radix (Niuxi), Rheum officinale (Dahuang), Glycyrrhiza uralensis (Gancao)
Zhang et al. (2016)	Touxie Jiedu Zhiyang Decoction	Astragalus membranaceus (Huangqi), Scutellaria baicalnsis (Huangqin), Rehmannia glutinosa (Dihuang), Paeonia lactiflora (Baishao), Angelicae Sinensis (Danggui), Dipsaci Radix (Xuduan), Persicae Semen (Taoren), Eucommia ulmoides (Duzhong), Codonopsis pilosula (Dangshen), Chaenomelis Fructus (Mugua), Carthami Flos (Honghua), Spatholobi Caulis (Jixueteng), Clematidis Radix (Weilingxian), Angelicae Dahuricae Radix (Baizhi), Achyranthis Bidentatae Radix (Niuxi), Rheum officinale (Dahuang), Glycyrrhiza uralensis (Gancao)
Diao et al. (2018)	Touxie Jiedu Zhiyang Decoction	Astragalus membranaceus (Huangqi), Scutellaria baicalnsis (Huangqin), Rehmannia glutinosa (Dihuang), Paeonia lactiflora (Baishao), Angelicae Sinensis (Danggui), Dipsaci Radix (Xuduan), Persicae Semen (Taoren), Eucommia ulmoides (Duzhong), Codonopsis pilosula (Dangshen), Chaenomelis Fructus (Mugua), Carthami Flos (Honghua), Spatholobi Caulis (Jixueteng), Clematidis Radix (Weilingxian), Angelicae Dahuricae Radix (Baizhi), Achyranthis Bidentatae Radix (Niuxi), Rheum officinale (Dahuang), Glycyrrhiza uralensis (Gancao)
Shi (2019)	Touxie Jiedu Zhiyang Decoction	Astragalus membranaceus (Huangqi), Scutellaria baicalnsis (Huangqin), Rehmannia glutinosa (Dihuang), Paeonia lactiflora (Baishao), Angelicae Sinensis (Danggui), Dipsaci Radix (Xuduan), Persicae Semen (Taoren), Eucommia ulmoides (Duzhong), Codonopsis pilosula (Dangshen), Chaenomelis Fructus (Mugua), Carthami Flos (Honghua), Spatholobi Caulis (Jixueteng), Clematidis Radix (Weilingxian), Angelicae Dahuricae Radix (Baizhi), Achyranthis Bidentatae Radix (Niuxi), Rheum officinale (Dahuang), Glycyrrhiza uralensis (Gancao)
Chen (2020)	Touxie Jiedu Zhiyang Decoction	Astragalus membranaceus (Huangqi), Scutellaria baicalnsis (Huangqin), Rehmannia glutinosa (Dihuang), Paeonia lactiflora (Baishao), Angelicae Sinensis (Danggui), Dipsaci Radix (Xuduan), Persicae Semen (Taoren), Eucommia ulmoides (Duzhong), Codonopsis pilosula (Dangshen), Chaenomelis Fructus (Mugua), Carthami Flos (Honghua), Spatholobi Caulis (Jixueteng), Clematidis Radix (Weilingxian), Angelicae Dahuricae Radix (Baizhi), Achyranthis Bidentatae Radix (Niuxi), Rheum officinale (Dahuang), Glycyrrhiza uralensis (Gancao)
Liu (2015)	Yangxue Runfu Yin	Astragalus membranaceus (Huangqi), Angelicae Sinensis (Danggui), Rehmanniae Radix (Dihuang), Kochia scoparia (Difuzi), Scutellaria baicalnsis (Huangqin), Asparagus cochinchinensis (Tianmendong), Ophiopogon japonicus (Maidong), Persicae Semen (Taoren), Carthami Flos (Honghua), Trichosanthis Radix (Tianhuafen), Cimicifuga dahurica (Shengma)
Hu (2019)	Yangxue Runfu Yin	Astragalus membranaceus (Huangqi), Angelicae Sinensis (Danggui), Rehmanniae Radix (Dihuang), Kochia scoparia (Difuzi), Scutellaria baicalnsis (Huangqin), Asparagus cochinchinensis (Tianmendong), Ophiopogon japonicus (Maidong), Persicae Semen (Taoren), Carthami Flos (Honghua), Trichosanthis Radix (Tianhuafen), Cimicifuga dahurica (Shengma)
Wang et al. (2019)	Yangxue Runfu Yin	Astragalus membranaceus (Huangqi), Angelicae Sinensis (Danggui), Rehmanniae Radix (Dihuang), Kochia scoparia (Difuzi), Scutellaria baicalnsis (Huangqin), Asparagus cochinchinensis (Tianmendong), Ophiopogon japonicus (Maidong), Persicae Semen (Taoren), Carthami Flos (Honghua), Trichosanthis Radix (Tianhuafen), Cimicifuga dahurica (Shengma)
Dou (2021)	Yangxue Runfu Yin	Astragalus membranaceus (Huangqi), Angelicae Sinensis (Danggui), Rehmanniae Radix (Dihuang), Kochia scoparia (Difuzi), Scutellaria baicalnsis (Huangqin), Asparagus

		cochinchinensis (Tianmendong), Ophiopogon japonicus (Maidong), Persicae Semen (Taoren), Carthami Flos (Honghua), Trichosanthis Radix (Tianhuafen), Cimicifuga dahurica (Shengma)
Zhu et al. (2004)	Yangxue Wensheng Decoction	Aconiti Lateralis Radix Praeparata (Fuzi), Cinnamomum cassia (Rougui), Rehmanniae Radix (Shengdihuang), Polygonum multiflorum (Heshouwu), Angelicae Sinensis (Danggui), Ligusticum chuanxiong (Chuanxiong), Salviae Miltiorrhizae (Danshen), Paeonia anomala (Chishao), Carthami Flos (Honghua), Codonopsis pilosula (Dangshen), Cuscuta chinensis (Tusizi), Schizonepetae Herba (Jingjie), Atractylodis Rhizoma (Cangzhu), Phellodendron amurense (Huangbai), Glycyrrhiza uralensis (Gancao)
He (2006)	Modified Siwu Tang and Erzhi Wan	Angelicae Sinensis (Danggui), Paeonia lactiflora Pall. (Baishao), Rehmanniae Radix (Shengdihuang), Ligusticum chuanxiong (Chuanxiong), Fructus Ligustri Lucidi (Nuzhenzi), Eclipta prostrata (Mohanlian), Dictamni Cortex.(Baixianpi), Kochiae Fructus (Difuzi)
Wang et al. (2013)	Xiaoyang Particles	Schizonepetae Herba (Jingjie), Saposhnikovia divaricata (Fangfeng), Cicadae Periostracum (Chantui), Anemarrhena asphodeloides (Zhimu), Gypsum Fibrosum (Shigao), Atractylodis Rhizoma (Cangzhu), Sophora flavescens (Kushen), Angelicae Sinensis (Danggui), Pheretima (Dilong), Rehmanniae Radix (Shengdihuang), Cannabis Fructus (Huomaren), Glycyrrhiza uralensis (Gancao), Rheum officinale (Dahuang)
Ge (2018)	Xiaofeng Zhiyang Particles	Schizonepetae Herba (Jingjie), Saposhnikovia divaricata (Fangfeng), Cicadae Periostracum (Chantui), Angelicae Sinensis (Danggui), Rehmanniae Radix (Shengdihuang), Lycii Cortex (Digupi), Atractylodis Rhizoma (Cangzhu), Gypsum Fibrosum (Shigao), Akebiae Caulis (Guanmutong), Glycyrrhiza uralensis (Gancao)
Tang et al. (2018)	Zhiyang Decoction	Schizonepetae Herba (Jingjie), Saposhnikovia divaricata (Fangfeng), Angelicae Sinensis (Danggui), Polygonum multiflorum (Heshouwu), Rehmanniae Radix (Shengdihuang), Morus alba (Sangye), Sophora flavescens (Kushen), Bombyx Batryticatus (Jiangcan), Cicadae Periostracum (Chantui), Sepiae Endoconcha (Haipiaoxiao), Ostreae Concha (Muli), Fossilia Ossia Mastodi (Longgu), Pueraria lobata (Gegen), Rheum officinale (Dahuang), Salviae Miltiorrhizae (Danshen)
Liu et al. (2019)	Jingfu Zhiyang Particles	Schizonepetae Herba (Jingjie), Saposhnikovia divaricata (Fangfeng), Chrysanthemum morifolium (Juhua), Houttuynia cordata (Yuxingcao), Kochiae Fructus (Difuzi), Crataegi Fructus (Shanzha), Poria cocos (Fuling)
Fan (2020)	Touxie Jiedu Zhiyang Decoction	Spatholobi Caulis (Jixueteng), Angelicae Sinensis (Danggui), Smilacis Glabrae Rhizoma (Tufuling), Rehmanniae Radix (Shengdihuang), Dioscoreae Rhizoma (Shanyao), Clematidis Radix (Weilingxian), Polistes mandarinus Saussure (Fengfang)
Yang et al. (2020)	Baifuzi Weiliang Decoction	Typhonii Rhizoma (Baifuzi), Atractylodis Rhizoma (Cangzhu), Coptis chinensis (Chuanhuanglian), Sophora flavescens (Kushen), Schizonepetae Herba (Jingjie), Saposhnikovia divaricata (Fangfeng), Cicadae Periostracum (Chantui), Bombyx Batryticatus (Jiangcan), Cnidium monnieri (Shechuangzi), Xanthium strumarium (Cangerzi), Arctii Fructus (Niubangzi), Rehmanniae Radix (Shengdihuang), Tribulus terrestris (Jili), Lonicerae Japonicae Flos (Jinyinhua)
Zhao (2020)	Siwu Decoction	Rehmannia glutinosa (Shoudihuang), Angelicae Sinensis (Danggui), Paeonia lactiflora Pall. (Baishao), Ligusticum chuanxiong (Chuanxiong)
Jin et al. (2021)	Modified Mahuang Lianqiao Chixiaodou Decoction and Yiyifuzhi Baijiang Decoction	Ephedra sinica Stapf (Mahuang), Forsythia suspensa (Lianqiao), Vigna umbellate (Chixiaodou), Amygdalus Communis (Xingren), Mori Cortex (Sangbaipi), Coicis Semen (Yiyiren), Aconiti Lateralis Radix Praeparata (Fuzi), Patrinia villosa (Baijiang), Glycyrrhiza uralensis (Gancao), Zingiberis Rhizoma Recens (Shengjiang), Jujubae Fructus (Dazao), Angelicae Sinensis (Danggui)
Wang et al. (2021)	Feng Xueqing Yin	Rehmanniae Radix (Shengdihuang), Angelicae Sinensis (Danggui), Paeonia lactiflora Pall. (Baishao), Ligusticum chuanxiong (Chuanxiong), Scrophularia ningpoensis (Xuanshen), Anemarrhena asphodeloides (Zhimu), Schizonepetae Herba (Jingjie), Saposhnikovia divaricata (Fangfeng)
Wu et al. (2021)	Modified Qufeng Decoction	Polygoni Multiflori Caulis (Shouwuteng), Dictamni Cortex.(Baixianpi), Spatholobi Caulis (Jixueteng), Cicadae Periostracum (Chantui), Saposhnikovia divaricata (Fangfeng), Glycyrrhiza uralensis (Gancao), Uncaria rhynchophylla (Gouteng), Tribulus terrestris L. (Jili), Kochiae Fructus (Difuzi), Sinomenii Caulis (Qingfengteng), Cnidium monnieri (Shechuangzi), Schizonepetae Herba (Jingjie), Paeonia suffruticosa Andr. (Mudanpi), Carthami Flos (Honghua)
Zhou et al. (2021)	Chinese herbal medicine	Astragalus membranaceus (Huangqi), Angelicae Sinensis (Danggui), Salviae Miltiorrhizae (Danshen), Paeonia lactiflora Pall. (Baishao), Atractylodes macrocephala (Baizhu), Dictamni Cortex.(Baixianpi), Kochiae Fructus (Difuzi), Ligusticum chuanxiong (Chuanxiong), Smilacis Glabrae Rhizoma (Tufuling), Schizonepetae Herba (Jingjie), Saposhnikovia divaricata (Fangfeng)

Wang et al. (2010)	Jiebiao Qufengzhiyang particles	Cinnamomi Ramulus (Guizhi), Paeonia lactiflora Pall. (Baishao), Astragalus membranaceus (Huangqi), Angelicae Sinensis (Danggui), Schizonepetae Herba (Jingjie), Saposhnikovia divaricata (Fangfeng), Ostreae Concha (Muli), Fossilia Ossia Mastodi (Longgu), Mume Fructus (Wumei), Glycyrrhiza uralensis (Gancao), Dictamni Cortex.(Baixianpi), Kochiae Fructus (Difuzi)
Luo et al. (2010)	Chinese herbal medicine	Astragalus membranaceus (Huangqi), Angelicae Sinensis (Danggui), Codonopsis pilosula (Dangshen), Paeonia lactiflora Pall. (Baishao), Paeonia anomala (Chishao), Persicae Semen (Taoren), Carthami Flos (Honghua), Sophora flavescens (Kushen), Smilacis Glabrae Rhizoma (Tufuling), Dictamni Cortex.(Baixianpi), Kochiae Fructus (Difuzi), Poria cocos (Fuling), Rheum officinale (Dahuang), Glycyrrhiza uralensis (Gancao), Zaocys dhumnades (Wuchiaoshe)
Zhang et al. (2011)	Qingxin Lianxi Yin	Nelumbinis Plumula (Lianzi), Ophiopogon japonicus (Maidong), Angelicae Sinensis (Danggui), Polygonum multiflorum (Heshouwu), Astragalus membranaceus (Huangqi), Codonopsis pilosula (Dangshen), Scutellaria baicalnsis (Huangqin), Lycii Cortex (Digupi), Schizonepetae Herba (Jingjie), Saposhnikovia divaricata (Fangfeng), Poria cocos (Fuling), Glycyrrhiza uralensis (Gancao)
Li (2015)	Modified Siwu Decoction	Astragalus membranaceus (Huangqi), Rehmannia glutinosa (Shoudihuang), Angelicae Sinensis (Danggui), Ligusticum chuanxiong (Chuanxiong), Vincetoxicum paniculatum (Xuchangqing), Asarum sieboldii (Xixin), Alismatis Rhizoma (Zexie), Paeonia anomala (Chishao), Gastrodia elata (Tianma), Polygonum multiflorum (Heshouwu)
Wang et al. (2016)	Shengyang Xiehuo Decoction	Bupleurum chinens (Chaihu), Astragalus membranaceus (Huangqi), Paeonia lactiflora (Baishao), Notopterygium incisum (Qianghuo), Cimicifuga dahurica Maxim. (Shengma), Codonopsis pilosula (Dangshen), Angelicae Sinensis (Danggui), Rheum officinale (Dahuang), Salviae Miltiorrhizae (Danshen), Achyranthes bidentata (Niuxi), Coptis chinensis (Huanglian), Cnidium monnieri (Shechuangzi), Dictamni Cortex.(Baixianpi), Kochiae Fructus (Difuzi)
Wu (2016)	Jiawei Jiedu Huoxue Decoction	Forsythia suspensa (Lianqiao), Persicae Semen (Taoren), Carthami Flos (Honghua), Glycyrrhiza uralensis (Gancao), Poncirus trifoliata (Zhiqiao), Pueraria lobata Ohwi (Yege), Angelicae Sinensis (Danggui), Paeonia anomala (Chishao), Rehmanniae Radix (Shengdihuang), Bupleurum chinens (Chaihu), Schizonepetae Herba (Jingjie), Dictamni Cortex.(Baixianpi), Kochiae Fructus (Difuzi), Cicadae Periostracum (Chantui)
Xie (2016)	Modified Siwu Decoction	Rehmannia glutinosa (Shoudihuang), Angelicae Sinensis (Danggui), Paeonia anomala (Chishao), Ligusticum chuanxiong (Chuanxiong), Phellodendron amurense Rupr. (Huangbo), Salviae Miltiorrhizae (Danshen), Cnidium monnieri (Shechuangzi), Kochiae Fructus (Difuzi), Asarum sieboldii (Xixin), Alismatis Rhizoma (Zexie), Polygonum multiflorum (Heshouwu), Astragalus membranaceus (Huangqi)
Li and Hong et al. (2019)	Danggui Yinzi	Angelicae Sinensis (Danggui), Paeonia lactiflora Pall. (Baishao), Ligusticum chuanxiong (Chuanxiong), Rehmanniae Radix (Shengdihuang), Tribulus terrestris (Jili), Schizonepetae Herba (Jingjie), Saposhnikovia divaricata (Fangfeng), Polygonum multiflorum (Heshouwu), Astragalus membranaceus (Huangqi), Rheum officinale (Dahuang), Dioscorea opposita Thunb. (Shanyao), Sophora flavescens (Kushen), Atractylodes chinensis (Cangzhu), Glycyrrhiza uralensis (Gancao)
Wu (2019)	Wushe Rongpi Decoction	Zaocys dhumnades (Wuchiaoshe), Rehmanniae Radix (Shengdihuang), Angelicae Sinensis (Danggui), Cinnamomi Ramulus (Guizhi), Paeonia anomala (Chishao), Ligusticum chuanxiong (Chuanxiong), Persicae Semen (Taoren), Carthami Flos (Honghua), Paeonia suffruticosa Andr. (Mudanpi), Lithospermum erythrorhizon (Zicao), Polygonum multiflorum (Heshouwu), Dictamni Cortex.(Baixianpi), Tribulus terrestris (Jili), Glycyrrhiza uralensis (Gancao) , Zingiberis Rhizoma Recens (Shengjiang), Jujubae Fructus (Dazao)
Hsu (2020)	Modified Danggui Yinzi	Angelicae Sinensis (Danggui), Paeonia lactiflora Pall. (Baishao), Ligusticum chuanxiong (Chuanxiong), Rehmanniae Radix (Shengdihuang), Tribulus terrestris (Jili), Schizonepetae Herba (Jingjie), Saposhnikovia divaricata (Fangfeng), Astragalus membranaceus (Huangqi), Kochiae Fructus (Difuzi), Glycyrrhiza uralensis (Gancao), Sophora flavescens (Kushen), Lycii Cortex (Digupi), Morus alba (Sangbaipi)
Li et al. (2020)	Mahuang Lianqiao Chixiaodou Decoction	Ephedra sinica Stapf (Mahuang), Forsythia suspensa (Lianqiao), Vigna umbellate (Chixiaodou), Amygdalus Communis (Xingren), Mori Cortex (Sangbaipi), Glycyrrhiza uralensis (Gancao), Zingiberis Rhizoma Recens (Shengjiang), Jujubae Fructus (Dazao),
Wong (2021)	Taohong Danggui Yinzi	Persicae Semen (Taoren), Carthami Flos (Honghua), Angelicae Sinensis (Danggui), Paeonia lactiflora Pall. (Baishao), Ligusticum chuanxiong (Chuanxiong), Rehmanniae Radix (Shengdihuang), Tribulus terrestris (Jili), Schizonepetae Herba (Jingjie), Saposhnikovia divaricata (Fangfeng), Astragalus membranaceus (Huangqi), Glycyrrhiza uralensis (Gancao), Polygonum multiflorum (Heshouwu)

Ren (2022)	Xiaofeng Zhiyang granules	Schizonepetae Herba (Jingjie), Saposhnikovia divaricata (Fangfeng), Cicadae Periostracum (Chantui), Angelicae Sinensis (Danggui), Rehmanniae Radix (Shengdihuang), Lycii Cortex (Digupi), Atractylodis Rhizoma (Cangzhu), Gypsum Fibrosum (Shigao), Akebiae Caulis (Guanmutong), Glycyrrhiza uralensis (Gancao)
Liu (2013)	Buyanghuanwu Tang	Astragalus membranaceus (Huangqi), Angelicae Sinensis (Danggui), Paeonia anomala (Chishao), Ligusticum chuanxiong (Chuanxiong), Persicae Semen (Taoren), Carthami Flos (Honghua), Pheretima (Dilong)
Lu (2015)	Modified Jiedu Zhiyang Decoction	Angelicae Sinensis (Danggui), Spatholobi Caulis (Jixueteng), Dictamni Cortex.(Baixianpi), Kochiae Fructus (Difuzi)
Zhao (2018)	Qingjiangxiezhuo decoction	Rheum officinale (Dahuang), Astragalus membranaceus (Huangqi), Rehmanniae Radix (Shengdihuang), Poria cocos (Fuling), Citri Reticulatae Pericarpium (Chenpi), Perilla frutescens (L.) Britt. (Suye), Perilla frutescens (L.) Britt (Sugeng), Eupatorium fortunei Turcz. (Peilian), Pueraria lobata (Gegen), Ligusticum chuanxiong (Chuanxiong), Angelicae Sinensis (Danggui), Kochiae Fructus (Difuzi), Glycyrrhiza uralensis (Gancao)
Zhang (2019)	Yishen Huoxue Decoction	Astragalus membranaceus (Huangqi), Angelicae Sinensis (Danggui), Salviae Miltiorrhizae (Danshen), Carthami Flos (Honghua), Rheum officinale (Dahuang), Atractylodes macrocephala (Baizhu), Poria cocos (Fuling), Schizonepetae Herba (Jingjie), Saposhnikovia divaricata (Fangfeng), Dictamni Cortex.(Baixianpi), Kochiae Fructus (Difuzi), Cicadae Periostracum (Chantui), Panax notoginseng (Sanqi)

Table S3. Grade profile summary of 'Chinese Herbal Medicine for uremic pruritus' Quality assessment

Certainty assessment							№ of patients		Effect		Certainty	Importance
№ of studies	Study design	Risk of bias	Inconsistency	Indirectness	Imprecision	Other considerations	Chinese herbal medicine	control	Relative (95% CI)	Absolute (95% CI)		
Overall effectiveness												
37	randomised trials	serious	not serious	not serious	not serious	publication bias strongly suspected	1070/1232 (86.9%)	792/1226 (64.6%)	RR 1.29 (1.23 to 1.35)	187 more per 1,000 (from 149 more to 226 more)	⊕⊕◯◯ Low	CRITICAL
Visual analog scale (VAS)												
21	randomised trials	serious	serious	not serious	not serious	publication bias strongly suspected	815	810	-	MD 1.98 lower (2.23 lower to 1.73 lower)	⊕◯◯◯ Very low	CRITICAL
PSQI												
3	randomised trials	serious	not serious	not serious	serious	None	93	94	-	MD 2.2 lower (2.77 lower to 1.64 lower)	⊕◯◯◯ Very low	CRITICAL
QOL												
4	randomised trials	serious	very serious	not serious	serious	None	137	137	-	MD 7.65 higher (2.71 higher to 12.59 higher)	⊕◯◯◯ Very low	CRITICAL

Certainty assessment							№ of patients		Effect		Certainty	Importance
№ of studies	Study design	Risk of bias	Inconsistency	Indirectness	Imprecision	Other considerations	Chinese herbal medicine	control	Relative (95% CI)	Absolute (95% CI)		
Adverse drug reaction (ADR)												
8	randomised trials	not serious	serious	not serious	not serious	None	20/258 (7.8%)	51/261 (19.5%)	RR 0.60 (0.22 to 1.63)	78 fewer per 1,000 (from 152 fewer to 123 more)	⨁⨁◯◯ Low	CRITICAL
Ca												
19	randomised trials	not serious	very serious	not serious	not serious	None	680	674	-	MD 0.01 higher (0.09 lower to 0.11 higher)	⨁◯◯◯ Very low	IMPORTANT
P												
26	randomised trials	not serious	very serious	not serious	not serious	None	868	862	-	MD 0.2 lower (0.28 lower to 0.13 lower)	⨁◯◯◯ Very low	IMPORTANT
PTH												
17	randomised trials	not serious	very serious	not serious	not serious	None	550	548	-	MD 76.68 lower (115.62 lower to 37.74 lower)	⨁◯◯◯ Very low	IMPORTANT

Certainty assessment							№ of patients		Effect		Certainty	Importance
№ of studies	Study design	Risk of bias	Inconsistency	Indirectness	Imprecision	Other considerations	Chinese herbal medicine	control	Relative (95% CI)	Absolute (95% CI)		
iPTH												
6	randomised trials	not serious	very serious	not serious	not serious	None	222	224	-	MD 54.63 lower (109.67 lower to 0.42 higher)	⊕◯◯◯ Very low	IMPORTANT
Creatinine												
17	randomised trials	not serious	very serious	not serious	not serious	None	533	531	-	MD 52.31 lower (93.32 lower to 11.31 lower)	⊕◯◯◯ Very low	IMPORTANT
BUN												
15	randomised trials	not serious	very serious	not serious	not serious	None	488	486	-	MD 1.97 lower (3.67 lower to 0.26 lower)	⊕◯◯◯ Very low	IMPORTANT
eGFR												
2	randomised trials	not serious	not serious	not serious	serious	None	56	58	-	MD 2.82 higher (0.65 higher to 4.99 higher)	⊕⊕◯◯ Low	IMPORTANT

Certainty assessment							№ of patients		Effect		Certainty	Importance
№ of studies	Study design	Risk of bias	Inconsistency	Indirectness	Imprecision	Other considerations	Chinese herbal medicine	control	Relative (95% CI)	Absolute (95% CI)		
UA												
2	randomised trials	not serious	very serious	not serious	serious	None	75	75	-	MD 3.59 higher (49.45 lower to 56.63 higher)	⊕◯◯◯ Very low	IMPORTANT
CRP												
16	randomised trials	not serious	very serious	not serious	not serious	None	643	638	-	MD 1.9 lower (2.52 lower to 1.27 lower)	⊕◯◯◯ Very low	IMPORTANT
TNF-a												
2	randomised trials	not serious	not serious	not serious	serious	None	94	94	-	MD 16.88 lower (19.35 lower to 14.41 lower)	⊕⊕◯◯ Low	IMPORTANT
B2-MG												
12	randomised trials	not serious	very serious	not serious	not serious	None	462	459	-	MD 4.9 lower (6.78 lower to 3.02 lower)	⊕◯◯◯ Very low	IMPORTANT
IL-6												

Certainty assessment							№ of patients		Effect		Certainty	Importance
№ of studies	Study design	Risk of bias	Inconsistency	Indirectness	Imprecision	Other considerations	Chinese herbal medicine	control	Relative (95% CI)	Absolute (95% CI)		
4	randomised trials	not serious	very serious	not serious	not serious	None	159	159	-	MD 3.36 lower (5.26 lower to 1.45 lower)	⨁◯◯◯ Very low	IMPORTANT
Albumin												
6	randomised trials	not serious	very serious	not serious	not serious	None	231	231	-	MD 2.01 higher (0.19 lower to 4.22 higher)	⨁◯◯◯ Very low	IMPORTANT
Hb												
10	randomised trials	not serious	very serious	not serious	not serious	None	333	332	-	MD 4.52 higher (0.23 higher to 8.8 higher)	⨁◯◯◯ Very low	IMPORTANT
ALT												
4	randomised trials	not serious	not serious	not serious	serious	None	90	89	-	MD 0.11 lower (2.39 lower to 2.16 higher)	⨁⨁◯◯ Low	IMPORTANT
AST												

Certainty assessment							№ of patients		Effect		Certainty	Importance
№ of studies	Study design	Risk of bias	Inconsistency	Indirectness	Imprecision	Other considerations	Chinese herbal medicine	control	Relative (95% CI)	Absolute (95% CI)		
4	randomised trials	not serious	not serious	not serious	serious	None	90	89	-	MD 0.13 higher (1.7 lower to 1.96 higher)	⊕⊕◯◯ Low	IMPORTANT
K												
2	randomised trials	not serious	not serious	not serious	serious	None	46	44	-	MD 0.02 lower (0.17 lower to 0.14 higher)	⊕⊕◯◯ Low	IMPORTANT
Pruritis score (Duo's VAG/ Sergio)												
7	randomised trials	serious	very serious	not serious	not serious	None	211	215	-	MD 6.11 lower (8.28 lower to 3.94 lower)	⊕◯◯◯ Very low	IMPORTANT
Pruritis score (Kuypers)												
2	randomised trials	serious	not serious	not serious	serious	None	61	61	-	MD 5.12 lower (6.49 lower to 3.75 lower)	⊕◯◯◯ Very low	IMPORTANT

Table S4. Search Strategy

**PUBMED**
1. Chronic kidney disease OR kidney injury OR kidney failure OR chronic renal failure OR end-stage renal disease OR end stage renal disease OR dialysis OR hemodialysis OR peritoneal dialysis
2. Uremic OR Uremia OR uremias
3. #1 OR #2
4. Pruritus OR Pruritis OR itch* OR xerosis OR skin problems OR skin disorders
5. Chinese patent medicine OR Chinese patent drugs OR Medicine, Chinese Traditional OR chinese herbologia OR Chinese medicine OR Chinese material medical OR Chinese herbs OR Chinese herbal medicine OR Herbal Medicine OR Chin Tradit Pat Med
6. Formula
7. (Decoction OR Tang)
8. (Pill OR Wan)
9. San [Title/Abstract]
10. Granule
11. Pian
12. Powder
13. #3 AND #4 AND (#5 OR #6 OR #7 OR #8 OR #9 OR #10 OR #11 OR #12)
**EMBASE**
1. 'chronic kidney disease'/exp OR 'chronic kidney disease' OR (chronic AND ('kidney'/exp OR kidney) AND ('disease'/exp OR disease)) OR 'kidney injury'/exp OR 'kidney injury' OR (('kidney'/exp OR kidney) AND ('injury'/exp OR injury)) OR 'kidney failure'/exp OR 'kidney failure' OR (('kidney'/exp OR kidney) AND ('failure'/exp OR failure)) OR 'chronic renal failure'/exp OR 'chronic renal failure' OR (chronic AND ('renal'/exp OR renal) AND ('failure'/exp OR failure)) OR 'end-stage renal disease'/exp OR 'end-stage renal disease' OR ('end stage' AND ('renal'/exp OR renal) AND ('disease'/exp OR disease)) OR 'end stage renal disease'/exp OR 'end stage renal disease' OR (end AND stage AND ('renal'/exp OR renal) AND ('disease'/exp OR disease)) OR 'dialysis'/exp OR dialysis OR 'hemodialysis'/exp OR hemodialysis OR 'peritoneal dialysis'/exp OR 'peritoneal dialysis' OR (peritoneal AND ('dialysis'/exp OR dialysis))
2. uremic OR 'uremia'/exp OR uremia OR uremias
3. #1 OR #2
4. 'pruritus'/exp OR pruritus OR 'pruritis'/exp OR pruritis OR itch* OR 'xerosis'/exp OR xerosis OR 'skin problems' OR (('skin'/exp OR skin) AND problems) OR 'skin disorders' OR (('skin'/exp OR skin) AND ('disorders'/exp OR disorders))
5. 'chinese medicine'/exp OR 'chinese medicine' OR 'herbal medicine'/exp OR 'herbal medicine' OR 'chinese patent medicine':ti,ab,kw OR 'chinese patent drugs':ti,ab,kw OR 'chinese herbologia':ti,ab,kw OR 'medicine, chinese traditional':ti,ab,kw OR 'chinese material medical':ti,ab,kw OR 'chinese herbs':ti,ab,kw OR 'chinese herbal medicine':ti,ab,kw OR 'chin tradit pat med':ti,ab,kw
6. ('formula'/exp OR formula)
7. ('decoction'/exp OR decoction OR tang)
8. ('pill'/exp OR pill OR wan)
9. san:ti,ab,kw
10. ('granule'/exp OR granule)
11. ('pian'/exp OR pian)
12. ('powder'/exp OR powder)
13. #3 AND #4 AND (#5 OR #6 OR #7 OR #8 OR #9 OR #10 OR #11 OR #12)
**Cumulative Index to Nursing and Allied Health Literature (CINAHL)**
1. (chronic kidney disease OR kidney injury OR kidney failure OR chronic renal failure OR end-stage renal disease OR end stage renal disease OR dialysis OR hemodialysis OR peritoneal dialysis)
2. (uremic OR Uremia OR uremias)
3. #1 OR #2
4. (Pruritus OR Pruritis OR itch* OR xerosis OR skin problems OR skin disorders)
5. (Chinese patent medicine OR Chinese patent drugs OR traditional Chinese medicine OR chinese herbologia OR Chinese medicine OR Chinese material medical OR Chinese herbs OR Chinese herbal medicine OR herbal medicine OR Chin Tradit Pat Med)
6. Formula
7. (Decoction OR Tang)

8. (Pill OR Wan)
9. Granule
10. Pian
11. Powder
12. San
13. #3 AND #4 AND (#5 OR #6 OR #7 OR #8 OR #9 OR #10 OR #11 OR #12)

Cochrane Central Register of Controlled Trials

1. (chronic kidney disease OR kidney injury OR kidney failure OR chronic renal failure OR end-stage renal disease OR end stage renal disease OR dialysis OR hemodialysis OR peritoneal dialysis)
2. (uremic OR Uremia OR uremias)
3. #1 OR #2
4. (Pruritus OR Pruritis OR itch* OR xerosis OR skin problems OR skin disorders)
5. (Chinese patent medicine OR Chinese patent drugs OR traditional Chinese medicine OR chinese herbologia OR Chinese medicine OR Chinese material medical OR Chinese herbs OR Chinese herbal medicine OR herbal medicine OR Chin Tradit Pat Med)
6. Formula
7. (Decoction OR Tang)
8. (Pill OR Wan)
9. Granule
10. Pian
11. Powder
12. San
13. #3 AND #4 AND (#5 OR #6 OR #7 OR #8 OR #9 OR #10 OR #11 OR #12)

Airiti library

1. Chronic kidney disease OR kidney failure OR dialysis OR hemodialysis OR peritoneal dialysis OR renal failure OR renal insufficiency OR membranous glomerulonephritis OR nephritis OR uremic OR Uremia OR uremias
2. Pruritus OR Pruritis OR itch* OR xerosis OR Skin Diseases
3. Chinese medicine OR chinese herbal medicine OR chinese and western medicine OR prescription OR (Decoction OR Tang) OR (Pill OR Wan) OR san OR formula OR powder OR granule OR pian OR jian
4. #1 AND #2 AND #3

China National Knowledge Infrastructure

1. Chronic kidney disease OR kidney failure OR dialysis OR hemodialysis OR peritoneal dialysis OR renal failure OR renal insufficiency OR membranous glomerulonephritis OR nephritis OR uremic OR Uremia OR uremias
2. Pruritus OR Pruritis OR itch* OR xerosis OR Skin Diseases
3. Chinese medicine OR chinese herbal medicine OR chinese and western medicine OR prescription OR (Decoction OR Tang) OR (Pill OR Wan) OR san OR formula OR powder OR granule OR pian OR jian
4. #1 AND #2 AND #3

Wangfang

1. Chronic kidney disease OR kidney failure OR dialysis OR hemodialysis OR peritoneal dialysis OR renal failure OR renal insufficiency OR membranous glomerulonephritis OR nephritis OR uremic OR Uremia OR uremias
2. Pruritus OR Pruritis OR itch* OR xerosis OR Skin Diseases
3. Chinese medicine OR chinese herbal medicine OR chinese and western medicine OR prescription OR (Decoction OR Tang) OR (Pill OR Wan) OR san OR formula OR powder OR granule OR pian OR jian
4. #1 AND #2 AND #3
